# STAT3 Cooperates With Phospholipid Scramblase 2 to Suppress Type I Interferon Response

**DOI:** 10.3389/fimmu.2018.01886

**Published:** 2018-08-15

**Authors:** Ming-Hsun Tsai, Chien-Kuo Lee

**Affiliations:** Graduate Institute of Immunology, College of Medicine, National Taiwan University, Taipei, Taiwan

**Keywords:** IFN-stimulated gene, palmitoylation, phospholipid scramblase 2, STAT3, type I interferon

## Abstract

Type I interferon (IFN-I) is a pluripotent cytokine that modulates innate and adaptive immunity. We have previously shown that STAT3 suppresses IFN-I response in a manner dependent on its N-terminal domain (NTD), but independent of its DNA-binding and transactivation ability. Using the yeast two-hybrid system, we have identified phospholipid scramblase 2 (PLSCR2) as a STAT3 NTD-binding partner and a suppressor of IFN-I response. Overexpression of PLSCR2 attenuates ISRE-driven reporter activity, which is further aggravated by co-expression of STAT3. Moreover, PLSCR2 deficiency enhances IFN-I-induced gene expression and antiviral activity without affecting the activation or nuclear translocation of STAT1 and STAT2 or the assembly of ISGF3 complex. Instead, PLSCR2 impedes promoter occupancy by ISGF3, an effect further intensified by the presence of STAT3. Moreover, palmitoylation of PLSCR2 is required for its binding to STAT3 and for this suppressive activity. In addition to STAT3, PLSCR2 also interacts with STAT2, which facilitates the suppressive effect on ISGF3-mediated transcriptional activity. Together, these results define the role of a novel STAT3–PLSCR2 axis in fine-tuning IFN-I response.

## Introduction

Type I interferon (IFN-I) is induced during the innate immune response and is required for initiating the antiviral response, growth inhibition, and immunomodulation (1). When IFN-I binds to IFNAR1 and IFNAR2 receptor complex, receptor-associated JAK kinases, namely, JAK1 and TYK2, are activated, leading to activation of STAT1 and STAT2 by tyrosine phosphorylation. IRF9 associates with activated STAT1 and STAT2 to form ISGF3, which translocates into the nucleus and initiates transcription of IFN-stimulated genes (ISGs) (2). STAT3 is also activated by IFN-I; however, unlike STAT1 and STAT2, STAT3 is a negative regulator of IFN-I response (3, 4), as the absence of STAT3 enhances IFN-I-mediated reporter activity, ISG induction, and antiviral response. The suppressive effect of STAT3 is independent of tyrosine phosphorylation, nuclear translocation, DNA-binding, and transactivation ability of the activated STAT1 and STAT2, as the N-terminal domain (NTD) of STAT3 is sufficient to confer its effects (4). The STAT3 NTD is involved in dimerization of unphosphorylated STAT3 and tetramerization of activated STAT3 (5, 6). By interacting with non-STAT proteins such as importin the NTD mediates nuclear translocation and nucleocytoplasmic shuttling, as well as the recruitment of phosphatases, HATs, and HDACs (7–12). However, it remains unclear how STAT3 suppresses IFN-I response.

Phospholipid scramblase (PLSCR) 2 is one of five structurally related members of the PLSCR family. PLSCR1 was the first identified and cloned and was named by its ability to redistribute phospholipid between inner and outer leaflets when reconstituted into proteoliposomes (13, 14). With the exception of the proline-rich domain (PRD), which is not conserved and may contribute to functional variety, PLSCR proteins share similar domain structures including a DNA-binding domain (DBD), palmitoylation motif (PAL), calcium-binding motif (CBD), nuclear localization signal (NLS), and transmembrane domain (TM) (15). Palmitoylation is a reversible posttranslational modification of proteins, which increases the hydrophobicity of proteins and facilitates their association with membranes or other proteins to affect their distribution and functions (16, 17). Depalmitoylated PLSCR1, PLSCR3, and PLSCR4 tend to localize to the nucleus, whereas phospholipid scramblase 2 (PLSCR2) predominantly remains in the nucleus regardless of its palmitoylation status (18–21). The palmitoylation motif-containing domain of PLSCR1 and PLSCR4 are known to be involved in protein–protein interactions (22, 23). Moreover, all of PLSCR proteins contain an atypical NLS and a DNA-binding motif, implying that PLSCR proteins may be imported into the nucleus and regulate gene expression (18, 24). However, the biological functions of the PLSCR family proteins remain to be determined. For example, although PLSCR1 was named after its scramblase activity, erythrocytes from PLSCR1KO mice show normal phosphatidylserine exposure (25), whereas the neutrophil numbers in both fetuses and newborns are significantly depressed, suggesting a role for this protein in immune cell homeostasis. Recently, PLSCR proteins were also reported to regulate innate immunity. PLSCR1 is an ISG known to interact with TLR9 and to partially regulate TLR9-induced IFN-I production through modulating TLR9 trafficking to endosomal compartments (26). Moreover, PLSCR1KO mouse embryonic fibroblasts (MEFs) display higher viral titers and lower ISG induction compared to WT and PLSCR1-restored MEFs after vesicular stomatitis virus (VSV) or encephalomyocarditis virus (EMCV) infection (27). Overexpression of PLSCR1 in HepG2 cells or mouse liver inhibits HBV replication *in vitro* and *in vivo*, respectively, suggesting that PLSCR1 is a positive regulator of IFN-I-induced antiviral responses (28). Nevertheless, the function of PLSCR2 remains largely unclear.

Here, we identified PLSCR2 as a STAT3-interacting partner and a negative regulator of IFN responses. Furthermore, the suppressive effect of PLSCR2 on this pathway appears to be STAT3 dependent. Thus, we define a novel STAT3–PLSCR2 axis that controls IFN-I and innate immune responses.

## Materials and Methods

### Cell Lines and Viruses

Mouse embryonic fibroblasts generated from STAT3^f/f^ mice were spontaneously immortalized through the standard 3T3 protocol. STAT3KO MEFs were generated by introducing Cre *via* adenoviral transduction into the STAT3^f/f^ MEFs *in vitro* (4). MEFs, ML-1 (29), Vero, and HEK293T cells were cultured in DMEM medium containing 10% fetal bovine serum (FBS; Biological Industries), and 50 µg/ml Gentamicin (Gibco). EMCV, VSV, and Sindbis virus (SINV)-GFP (30) and adenovirus virus (ADV)-GFP (31) were kindly provided by Dr. Lih-Hwa Hwang (National Yang-Ming University, Taiwan). Vaccinia virus (VV)-mCherry was kindly provided by Dr. Wen Chang (Academia Sinica, Taiwan) (32). PLSCR2KO ML-1 cell line was generated using the CRISPR-Cas9 system (33). In brief, ML-1 cells were cotransfected with one pair of vectors that carries Cas9 D10A nickase/EGFP and Cas9 D10A nickase/mCherry and sgRNAs targeting exon 3 of murine *Plscr2* (ENSMUST00000180154). The targeted sequences are 5′-GGTAGTTAGTCTGGAATGTG-3′ and 5′-GTTCCCCAGTCTGGTTATCC-3′, respectively (Zgenebio). After transfection for 24 h, EGFP/mCherry double-positive cells were sorted for single cells into 96-well plates. The clones were picked and screened by surveyor assay using T7 endonuclease I (NEB) for indel formation and western blotting for protein expression 2 weeks later.

### Antibodies and Cytokines

The antibodies and cytokines used were as follows: anti-α-tubulin (Epitomics), anti-phospho-STAT1 (Y701) (Abcam), anti-STAT1 (made in-house), anti-phospho-STAT2 (Y689) (Millipore), anti-STAT2 (made in-house), anti-phospho-STAT3 (Y705) (Epitomics), anti-STAT3 (Invitrogen), anti-IRF9 (Proteintech), anti-PLSCR2 (made in-house), anti-HA for immunoprecipitation and staining (made in-house), anti-HA for western blotting (clone: 12CA5), anti-myc (clone 9E10), recombinant human IFN-α2a (Roche), and recombinant murine IFN-α4 (made in-house).

### DNA Constructs

Full-length HA-tagged STAT3_1–770_, ΔN STAT3_135–770_, and NTD of STAT3_1–134_ were gifts from Dr. Joanna Jeou-Yuan Chen (Academia Sinica, Taiwan). Murine STAT1, STAT2, and IRF9 were amplified from cDNA and subcloned into pLPC-FH2, a vector carrying one flag and two HA tags, using *Xho*I, *Bam*HI/*Mfe*I, and *Bam*HI/*Eco*RI restriction sites, respectively. Full-length murine PLSCR2_1–307_ and truncated mutants PLSCR2_109–307_, PLSCR2_1–116_, PLSCR2_171–307_, and PLSCR2_188–307_ were generated by PCR amplification of a murine liver cDNA library and cloned into pCMV-myc using *EcoR*I and *Xho*I restriction sites. The palmitoylation-deficient CA mutant of PLSCR2 was generated by site-directed mutagenesis using the single primer method, as described (34). The primer sequence is 5′-CTGAAATGCAGTAGCGCCGCCTTCCCTGCAGCGCTCCAGGAGATAG-3′. The underlines indicate mutated nucleotides which change four conserved cysteine residues in the palmitoylation motif of PLSCR2 into four alanine residues. In brief, pCMV-myc-PLSCR2 was used as a template to amplify the mutation-containing DNA. The template construct was digested by *DpnI* to remove the parental unmutated strand of DNA and then transformed into an *E. coli* competent cell to recover the complementary strand. The CA mutant construct was mapped by newly generated *Pst*I restriction site and confirmed by sequencing.

### Quantitative Real-Time PCR

Total RNA was prepared using TRIsure™ reagent (Bioline). For qRT-PCR analysis, 3 µg of DNase-treated RNA was reverse transcribed with MMLV reverse transcriptase (Bionovas) in 1× reaction buffer at 42°C for 60 m, followed by inactivation of the reaction by incubating at 70°C for 10 m. The cDNA was then subjected to quantitative real-time PCR using the following primer sets and analyzed with PikoReal Real-Time PCR System (Thermo Fisher Scientific). *β*-*Actin*, forward 5′-AGGGAAATCGTGCGTGAC-3′, reverse 5′-GCTCGTTGCCAATAGTGATG-3′; *Rpl7*, forward 5′-TCAACAAGGCTTCAATTAACAT-3′, reverse 5′-CAATCAAGGAATTATCTGTCAA-3′; *Ifit1*, forward 5′-ACTATGAGAAGGCACTGAG-3′, reverse 5′-ACGAACAACAACAACAACA-3′; *Ifit3*, forward 5′-CGCCATGTTCCGCCTAGA-3′, reverse 5′-CCAGGAGAACTTTCAGGTACTGGTT-3′. The relative mRNA was normalized to *β-Actin* or *Rpl7*.

### Lentivirus-Mediated shRNA Delivery

All the plasmids for the lentivirus production were purchased from the National RNAi Core Facility, Academia Sinica, Taiwan. Production of recombinant viruses was performed following the instructions (http://rnai.genmed.sinica.edu.tw). Briefly, pCMV-Δ8.91, pMD-2G, and a lentiviral vector containing individual shRNA targeting sequence were transfected into HEK293T cells using Maestrofectin™ transfection reagent (Maestrogen). The viral supernatants were collected 48 h later. MEFs or ML-1 cells were transduced with the recombinant lentiviruses in the presence of polybrene (8 µg/ml) by spin infection (1,100 *g*, 90 m at room temperature). After spin infection, the cells were incubated at 37°C for 1 h before changing the medium and were then incubated for another 48 h followed by selection with puromycin (8–10 µg/ml) for 1 week. The targeted sequences for luciferase, PLSCR2, STAT2, and STAT3 are as follows: shLuc, 5′-CAAATCACAGAATCGTCGTAT-3′; shPLSCR2, GCCAAAGCTCACTCTTCAGAA; shSTAT2, 5′-AGTCACATGCTTCGGTATAAG-3′; shSTAT3, 5′-CCTAACTTTGTGGTTCCAGAT-3′.

### Immunoprecipitation and Western Blot Analysis

Total cell lysates were prepared by lysing cells in IP lysis buffer (300 mM NaCl, 50 mM HEPES, pH 7.6, 1.5 mM MgCl_2_, 10% glycerol, 1% Triton X-100, 10 mM NaPyrPO_4_, 20 mM NaF, 1 mM EGTA, 0.1 mM EDTA, 1 mM DTT, 1 mM PMSF, 1 mM Na_3_VO_4_, and 1× protease inhibitor cocktail) at 4°C for 15 m and clarified by centrifugation at 12,000 *g* for 10 min. For immunoprecipitation, protein A agarose (Roche) was conjugated with rabbit anti-HA antibody in TBST and incubated at 4°C for 4 h. The conjugated beads were mixed with samples in IP lysis buffer at 4°C overnight. The beads were washed with IP lysis buffer four times and eluted by boiling in 1× sample buffer. The cytoplasmic and nuclear extracts were prepared as described (35). Briefly, cells were first lysed in RSB-G40 buffer (10 mM Tris, pH 7.5, 10 mM KCl, 3 mM MgCl_2_, 10% glycerol, and 0.25% NP-40; with fresh protease inhibitor cocktail added) for 15 m on ice. After centrifugation at 12,000 *g* for 5 m, the supernatants were collected as cytoplasmic extracts. Nuclei were washed with RSBG40 three times and then resuspended in high-salt buffer (20 mM HEPES, pH 7.9, 420 mM NaCl, 1.5 mM EDTA, and 25% glycerol; with fresh protease inhibitor cocktail added) at 4°C for 30 m. After centrifugation at 12,000 *g* for 5 m, the supernatants were collected as nuclear extracts. For western blot analysis, equal amounts of samples were resolved in SDS-PAGE, followed by transferring to a PVDF membrane (Millipore) and blotting with the indicated antibodies.

### Reporter Activity Assay

HEK293T cells were transfected with Maestrofectin™ (Maestrogen) at a ratio of 1:1 (DNA to reagent) and MEFs or ML-1 cells were transfected with Turbofect™ (Thermo Fisher Scientific) at a ratio of 1:2 (DNA to reagent). For reporter activity assay, pISRE-Luc (Stratagene) and a transfection control pEGFP-N1 were cotransfected with STAT3 and/or PLSCR2 expressing plasmids at a ratio of 1:15:10 and diluted in serum-free DMEM. Following the transfection for 48 h, the cells were treated with human IFN-α2a (for HEK293T) or mouse IFN-α4 (for MEF and ML-1) for 8 h. The luciferase activity assay was carried out using the Dual-Glo^®^ Luciferase Assay System (Promega) and measured by Orion II luminometer (Berthold).

### Immunofluorescent Microscopy

WT MEFs or PLS2KO ML1 cells grown on glass coverslips were transfected myc-PLSCR2 for 24 h. Transfected cells were fixed with pre-chilled methanol (5 m at −-20°C) or 3% formaldehyde (10 m at RT) and permeabilized with 0.2% Triton X-100 for 10 m and then washed twice with ice-cold PBS. After blocking with 1% BSA for 1 h, the fixed cells stained with primary mouse antibody to myc and rabbit antibody to STAT3 for 1 h, washed three times with 1× PBS, and stained secondary antibodies to FITC-conjugated anti-mouse IgG (Jackson ImmunoResearch) and DyLight 594-conjugated anti-rabbit IgG (Jackson ImmunoResearch). Slides were mounted in mounting medium (DakoCytomation) with DAPI and monitored by confocal microscopy (Leica TCS SP5 and Carl Zeiss LSM880).

### Chromatin Immunoprecipitation (ChIP) Assay

Chromatin immunoprecipitation assay was performed as described (36). Briefly, ML-1 cells were fixed in 1.42% formaldehyde at room temperature for 15 m, followed by quenching with 125 mM glycine. After washing with cold PBS, cells were then resuspended in nuclei lysis buffer (50 mM Tris, 10 mM EDTA, 1% SDS, 1 mM PMSF, and 1× protease inhibitor cocktail) on ice for 10 m. The extracts were then sonicated with a Vibra-Cell VCX 130 sonicator (Sonics & Materials). The sonication conditions were 15 s on and 45 s off on ice for 20 cycles at 100% power output to shear DNA to ~200 to 400 bp. Protein A-Sepharose beads (Roche) were added to the cell lysates which were pre-incubated with the corresponding antibodies overnight at 4°C. After extensive washes with ChIP buffer (50 mM NaCl, 50 mM Tris–HCl, pH 7.5, 5 mM EDTA, 0.5% NP-40, 1% Triton X-100, 0.5 mM PMSF, 10 mM NaF, and 0.1 mM Na_3_VO_4_), the DNA-bound beads were reverse-crosslinked at 67°C for overnight. Protein were removed by incubating with 20 µg/ml proteinase K-containing buffer (TE buffer with 0.25% SDS) at 55°C for 4 h. DNA was recovered by phenol–chloroform extraction and ethanol precipitation and analyzed by qPCR using primers specific for the corresponding ISREs in the promoters of the indicated genes. Primer sequences are as follows: ISRE of *Ifit1* promoter, forward 5′-GTGGAGAATGCAGTAGGGCAAAC-3′, reverse 5′-GTCACACCAACTGGAAGCTCAGG-3′; ISRE of *Ifit3* promoter, forward 5′-AAGGTCTCAGTGGTAAGTT-3′, reverse 5′-CTCTGCTGCTTCTAAGGA-3′.

### Antiviral Assay and Titration of Viruses

For antiviral state assays, MEFs or ML-1 cells stably expressing control (shLuc) or PLSCR2-specific shRNA (shPLS2) were pretreated for 24 h with or without twofold serial dilution of IFN-α4 starting from 1,000 U/ml. EMCV or VSV at an MOI of 0.1 was added to the cells under serum-free conditions and incubated at 37°C for 1 h. The supernatant was then replaced with 10% FBS-containing medium. The medium was removed at 24 h post-infection, and the cells were fixed with 10% formaldehyde for 20 m at room temperature and then visualized with 0.1% crystal violet. Plaque formation assay was used to measure the viral titers. Briefly, Vero cells were first infected with 10-fold serial dilution of the viral supernatants in serum-free DMEM for 1 h, and then replaced with DMEM containing 2% FBS and 1.5% methylcellulose to immobilize viruses. The cells were fixed and visualized with crystal violet 48 h later to enumerate the plaques.

### Electrophoretic Mobility Shift Assay (EMSA)

Electrophoretic mobility shift assay was performed according to the manufacturer’s instructions for LightShift Chemiluminescent EMSA kit (Thermo Fisher Scientific). Briefly, a pair of complementary oligonucleotides derived from the ISRE of ISG15 promoter was biotinylated at the 5′ end prior to annealing. The sequences are as follows: 5′-CTCGGGAAAGGGAAACCGAAACTGAAGCC-3′, 5′-GGCTTCAGTTTCGGTTTCCCTTTCCCGAG-3′. Nuclear extracts prepared from ML-1 cells that were pretreated with or without mouse IFN-α4 for the indicated times were incubated with 200 fmol of biotinylated ISRE probe in 1× binding buffer [1 µg poly(dI:dC), 0.05% NP-40, 2.5% glycerol, and 5 mM MgCl_2_] for 20 m. For evaluating ISGF3 binding, HEK293T cells were transfected individually with empty vector (EV), HA-tagged STAT3, myc-tagged WT or CA mutant of PLSCR2 or cotransfected with human STAT1, STAT2, and IRF9 for 48 h and then treated with human IFN-α2 for 60 m. Nuclear extracts were prepared as described (35) by extraction with 0.38 M NaCl, dialyzed in modified buffer D (20 mM Hepes, pH 7.9, 100 mM KCI, 0.5 mM EDTA, and 10% glycerol) and stored at −70°C for binding assay. Supershift assays were performed by adding 1 µl of rabbit anti-STAT1 antiserum (made in-house) or anti-IRF9 (Proteintech) to the mixture. The protein–DNA complexes were resolved on a 6% native polyacrylamide gel in 0.5× TBE buffer prior to transferring to a positively charged nylon membrane (Millipore). The transferred DNA was then crosslinked to the membrane using a transilluminator (BD UVP) equipped with 302 nm bulbs for 12 m. Shifted bands containing biotinylated DNA probe were detected using horseradish peroxidase (HRP) conjugated-streptavidin and ECL (Thermo Fisher Scientific), and visualized with an X-ray film (Fujifilm).

### Palmitoylation Assay

Palmitoylation assay was performed using the acyl-biotin exchange method as described (37). In brief, HEK293T cells were transfected with myc-tagged WT or CA mutant of PLSCR2 for 48 h. To block reactive cysteines, cells were lysed in *N*-ethylmaleimide 50 mM (NEM, Sigma) containing cold IP lysis buffer. Following centrifugation at 12,000 *g* for 10 m, the supernatants were incubated with anti-myc antibody conjugated protein-G beads (Millipore) overnight at 4°C. The beads were rapidly washed in IP lysis buffer (pH 7.4) containing 10 mM NEM and then treated with or without 1 M hydroxylamine (HAM) for 1 h at room temperature to cleave palmitate from cysteines. After washing three times with pH-adjusted IP lysis buffer (pH 6.2), the beads were exposed to sulfydryl-specific biotinylating reagent containing 1 µM biotin-BMCC (Thermo Fisher Scientific) in IP lysis buffer (pH 6.2) for 2 h at 4°C. The biotinylated proteins were eluted and analyzed by western blotting using streptavidin-conjugated HRP and ECL.

### Statistical Analysis

All statistical analysis was performed with unpaired, two-tailed Student’s *t*-tests. Values of *p < 0.05, **p < 0.01, and ***p < 0.001 were considered statistically significant.

## Results

### Identification of STAT3 NTD-Interacting Proteins Using the Yeast Two-Hybrid System

We have previously shown that STAT3 negatively regulates IFN-I response in a manner dependent on its NTD but independent of its DNA-binding and transactivation ability (4). Since the STAT3 NTD is mainly involved in protein–protein interactions, we reasoned that it might exert regulatory activity through the recruitment of effector molecules. Therefore, we performed a yeast two-hybrid screen to identify novel proteins interacting with the STAT3 NTD. A flow chart of the screening process is shown in Figure S1A in Supplementary Material. Three different versions of STAT3 NTD were constructed as bait: BD-134 (1–134 aa), BD-134K (change from E to K at position 16) and, BD-317 (1–317 aa), and their autoactivity in the absence of prey proteins was tested. BD-134K is an earlier version of mouse STAT3 reported (38). While both BD-134 and BD317 exhibited autoactivity, BD-134K did not (Figure S1B in Supplementary Material). To further identify the regions involved in autoactivation of BD-134, we generated six truncated mutants: BD-124 (1–124 aa), BD-114 (1–114 aa), BD-104 (1–104 aa), BD-84 (1–84 aa), BD-64 (1–64 aa), and BD-10-134 (10–134 aa), and BD-10-134K (10–134 aa with an change of E to K at position 16) was used as a negative control (Figure S1C in Supplementary Material). We found that both deletion of the first 10 aa and change at E16 to K was capable of eliminating autoactivation of BD134. As a result, BD-134K was chosen as the bait for screening against a normalized mouse cDNA library. After screening ~2 × 10^6^ transformants, we identified several positive clones (Figure S1D in Supplementary Material). Among them CCT3 and LYN have already been reported to interact with STAT3 (39, 40) and thus, validate this screening strategy. Nine partial cDNAs from the candidate genes were cloned and expressed in HEK293T cells to validate their ability to interact with the STAT3 NTD (Figure S1E in Supplementary Material). Four out of nine prey fragments, ACY1, GNB1, GNB4, and PLSCR2, were further verified for their interacting specificity. As shown in Figure S1F in Supplementary Material, all four fragments were able to interact with full-length STAT3, but not STAT3ΔN, suggesting that they indeed specifically interact with the STAT3 NTD. ACY1 encodes aminoacylase 1, an enzyme involved in the catabolism and salvage of acylated amino acids (41). GNB1 and GNB4 encode heterotrimeric G protein subunit β1 and β4, respectively, which are involved in chemokine-induced signaling pathways (42), and PLSCR2 is a member of the family of phospholipid scramblases (13, 14). Of these, PLSCR2 was chosen for further study because PLSCR1, another member of the PLSCR family, has already been shown to regulate IFN-I-induced antiviral response (27).

### PLSCR2 Is a STAT3-Interacting Protein, an IFN-Inducible Gene, and Is Predominantly Located to the Nucleus

**Figure 1:**
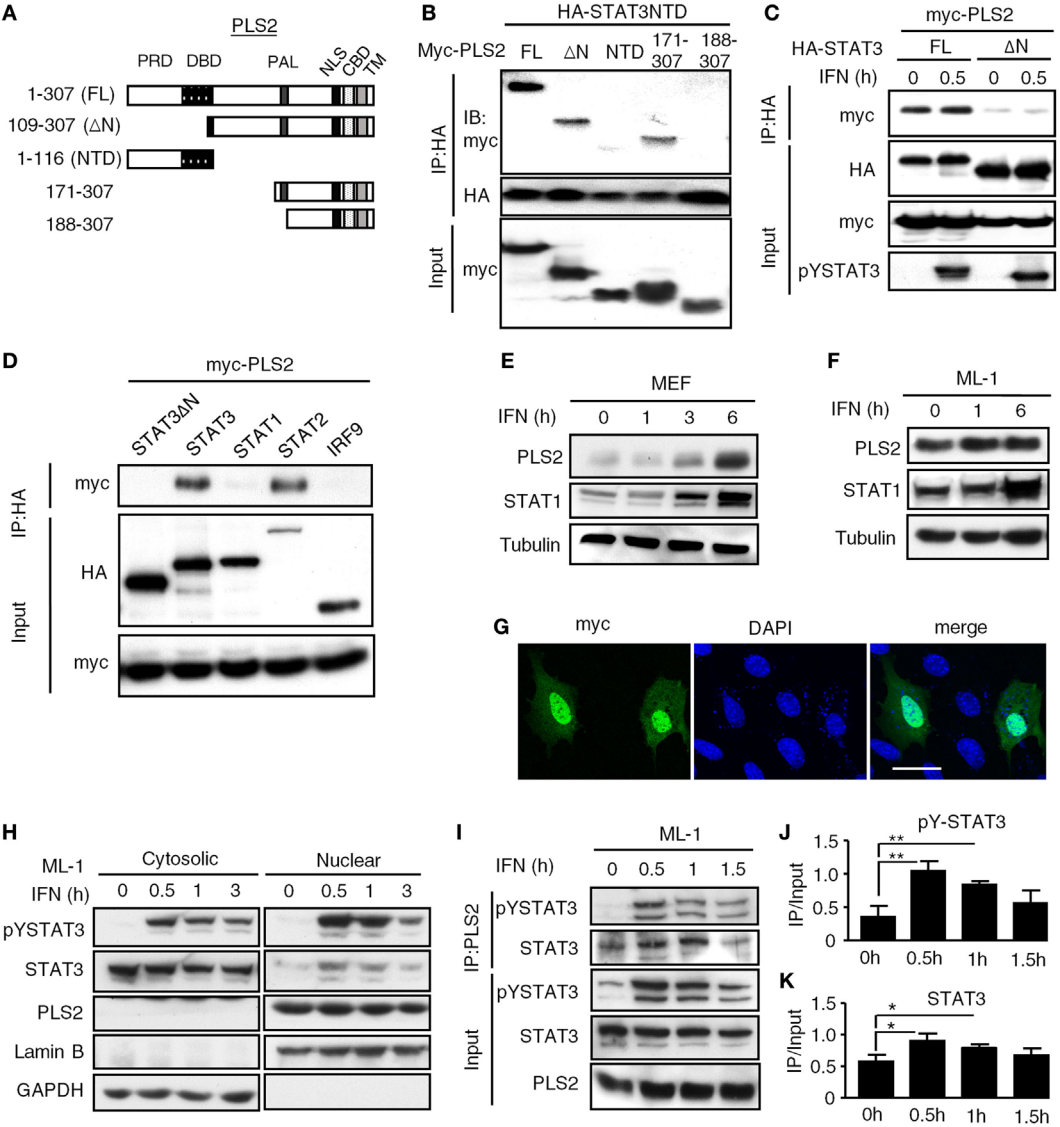
Phospholipid scramblase 2 (PLSCR2) is an IFN-inducible nuclear protein that interacts with the STAT3 N-terminal domain (NTD). **(A)** Schematic diagram of PLSCR2 full-length and truncated mutants. Abbreviations: PRD, proline-rich domain; DBD, DNA-binding domain; PAL, palmitoylation domain; NLS, nuclear localization signal; CBD, calcium-binding domain; TM, transmembrane domain. **(B)** HEK293T cells were transfected with HA-STAT3 NTD and myc-PLSCR2 (FL), PLSCR2_109–307_ (ΔN), PLSCR2_1–116_ (NTD), PLSCR2_171–307_, or PLSCR2_188–307_, followed by co-IP with anti-HA antibody and immunoblotting with antibodies against myc and HA, respectively. Total lysates subjected to immunoblotting with anti-myc antibody were used as the input control. Blots are representative of two independent experiments. **(C)** HEK293T cells were transfected with HA-STAT3 (FL) or STAT3_135–770_ (ΔN) and myc-PLSCR2 and then treated with human IFN-α2 (1,000 U/ml) for 30 m, followed by co-IP assay as described in panel **(B)**. Total lysates subjected to immunoblotting with anti-myc or pYSTAT3 antibody were used as the input control. Blots are representative of two independent experiments. **(D)** HEK293T cells were cotransfected with Myc-PLSCR2 and HA-tagged STAT3, STAT3_135–770_ (STAT3ΔN), STAT1, STAT2, or IRF9. The cell lysates were subjected to co-IP as in panel **(B)**. Total lysates subjected to immunoblotting with antibodies against HA or Myc were used as the input control. Blots are representative of two independent experiments. **(E,F)** WT mouse embryonic fibroblast (MEF) **(E)** or ML-1 **(F)** cells were treated with mouse IFN-α4 (1,000 U/ml) for the indicated times. Total cell lysates were subjected to immunoblotting with antibodies against PLSCR2, STAT1, or tubulin. Blots are representative of two independent experiments. **(G)** WT MEFs were transfected with myc-PLSCR2 for 24 h, fixed, and then stained with anti-myc antibody and FITC-conjugated anti-mouse IgG and DAPI. Samples were visualized by confocal microscopy. Images are representative three independent experiments. Scale bar = 25 µm. **(H)** ML-1 cells were stimulated with mouse IFN-α4 (1,000 U/ml) for the indicated times. Cytosolic and nuclear extracts were subjected to immunoblotting with antibodies against PLSCR2, phospho-STAT3, STAT3, lamin B, or GAPDH. Blots are representative of two independent experiments. **(I)** ML-1 cells stimulated with mouse IFN-α4 (1,000 U/ml) for the indicated times were immunoprecipitated with anti-PLSCR2 antibody and immunoblotted with antibodies against pSTAT3 and STAT3. Total cell lysates subjected to immunoblotting with antibodies against pYSTAT3, STAT3, and PLSCR2 were used as the input control. Blots are representative of three independent experiments. **(J,K)** Quantification of co-immunoprecipitated pY-STAT3 **(J)** and STAT3 **(K)** with PLSCR2 was done by normalizing to input signals using ImageJ. Data are shown as mean ± SD, *p < 0.05, **p < 0.01.

Sequencing analysis revealed that the prey fragment of PLSCR2 identified in the yeast two-hybrid screen encodes the C-terminal region of PLSCR2 (109–307 aa). We generated different truncation mutants of PLSCR2 (Figure 1A) and coexpressed these with the STAT3 NTD in HEK293T cells to dissect the interacting domains. As shown in Figure 1B, full-length (PLSCR2_1–307_), ΔN (PLSCR2_109–307_), and PLSCR2_171–307_, but not PLSCR2 NTD or PLSCR2_188–307_, were able to interact with the STAT3 NTD. Moreover, the interaction between PLSCR2 and STAT3 was severely abrogated in the absence of the STAT3 NTD (Figure 1C). This suggests that the STAT3 NTD is necessary and sufficient for interaction with PLSCR2, probably through the palmitoylation domain of PLSCR2. Moreover, the interactions were not affected by IFN-I treatment and thus, this activity is most likely constitutive (Figure 1C). We next assessed if PLSCR2 interacts with the components of ISGF3. Interestingly, in addition to STAT3, PLSCR2 also associates with STAT2, but not with STAT1 or IRF9 (Figure 1D). PLSCR2 was found to be expressed in different mouse cell lines, including MEFs and ML-1, a hepatoma cell line, although the basal levels of PLSCR2 were lower in MEFs than in ML-1 cells (Figures 1E,F). Moreover, PLSCR2 protein expression could be greatly induced by IFN-I treatment of MEFs and, to a lesser extent, of ML-1, suggesting that PLSCR2 is also an ISG.

To examine the intracellular localization of PLSCR2, we took advantage of the limited expression of PLSCR2 in MEF cells and used them to overexpress a myc-tagged PLSCR2. In these cells, the myc signal predominantly appeared in the nucleus even without cytokine stimulation (Figure 1G). The overexpressed PLSCR2 was also found to colocalize with endogenous STAT3, which was increased with IFN-I treatment, most likely due to nuclear enrichment of STAT3 (Figure S2B in Supplementary Material). Concomitantly, endogenous PLSCR2 was also mainly found in the nuclear fraction of ML-1 (Figure 1H) and MEF cells (Figure S2A in Supplementary Material) before and after IFN-I stimulation. The interaction between endogenous PLSCR2 and STAT3 was further confirmed by co-IP experiments. In the absence of IFN-I treatment, STAT3 was found to interact with PLSCR2 (Figure 1I). Following IFN-I treatment, the association of these proteins increased and then gradually decreased 1.5 h later, which coincides with the reduction of STAT3 in the nucleus (Figures 1J,K). These results suggest that the dynamic association between PLSCR2 and STAT3 may be dependent on the availability of nuclear STAT3 following IFN-I stimulation.

### PLSCR2 Suppresses IFN-I Response and Antiviral Activity

**Figure 2:**
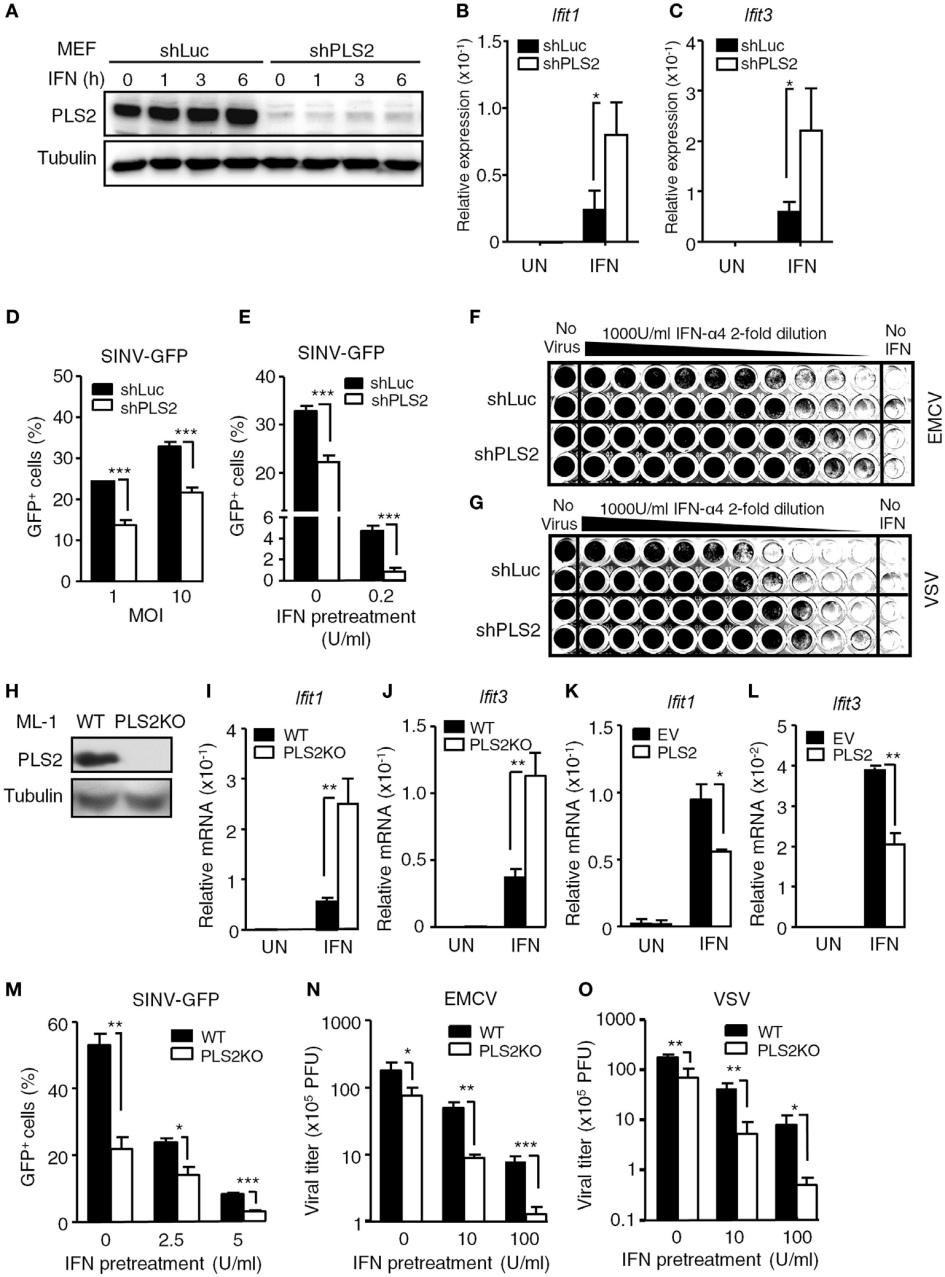
Phospholipid scramblase 2 (PLSCR2) suppresses IFN-induced IFN-stimulated gene (ISG) expression and antiviral responses. **(A)** WT mouse embryonic fibroblasts (MEFs) expressing luciferase- (shLuc) or PLSCR2-specific (shPLS2) shRNA were treated with or without IFN-α4 (1,000 U/ml) for the indicated times. Total cell lysates were subjected to immunoblotting with antibodies against PLSCR2 or tubulin. Blots are representative of two independent experiments. **(B,C)** Same as in panel **(A)**, except cells were treated with IFN-α4 for 6 h and RNA was subjected to RT-QPCR using primers for *Ifit1* **(B)**, *Ifit3* **(C)**, and *β-Actin*. Relative mRNA was normalized to *β-Actin* (*N* = 3). **(D)** shLuc or shPLS2 transduced WT MEFs were infected with Sindbis virus (SINV)-GFP at an MOI of 1 or 10 for 20 h followed by flow cytometric analysis for GFP^+^ cells (*N* = 4). **(E)** Same as in panel **(D)**, except the cells were pretreated with or without IFN-α4 (0.2 U/ml) for 24 h and then infected with SINV-GFP at an MOI of 1 (*N* = 4). **(F,G)** shLuc or shPLS2 transduced WT MEFs were pretreated with twofold serial dilution of IFN-α4 for 24 h before being infected with encephalomyocarditis virus (EMCV) **(F)** or vesicular stomatitis virus (VSV) **(G)** at an MOI of 1 for another 24 h. The viable cells were fixed and visualized with crystal violet. **(H)** Total cell lysates of WT and PLSCR2KO (PLS2KO) ML-1 cells were subjected to immunoblotting with antibodies against PLSCR2 or tubulin. **(I,J)** WT and PLS2KO ML-1 cells were treated with or without mouse IFN-α4 for 6 h. Total RNA was subjected to RT-QPCR using primers to *Ifit1* **(I)**, *Ifit3* **(J)**, and *Rpl7*. Relative mRNA was normalized to *Rpl7* (*N* = 7). **(K,L)** Same as in panel **(I)**, except PLS2KO ML-1 cells were transfected with empty vector (EV) or vector expressing PLSCR2 for 48 h (*N* = 3). **(M)** WT and PLS2KO ML-1 cells were treated with or without mouse IFN-α4 at 2.5 or 5 U/ml for 24 h and then infected with SINV-GFP at an MOI of 1 for 20 h followed by flow cytometric analysis for GFP^+^ cells (*N* = 3). **(N,O)** WT and PLS2KO ML-1 cells were pretreated with or without IFN-α4 at 10 or 100 U/ml for 24 h and then infected with EMCV **(N)** or VSV **(O)** at an MOI of 1 for 8 h. The viral titers were determined by plaque formation assay (*N* = 3). Data are shown as mean ± SD, *p < 0.05, **p < 0.01, and ***p < 0.001.

To investigate the role of endogenous PLSCR2 in IFN-I responses, MEFs were transduced with lentivirus expressing shRNA targeting PLSCR2 (shPLS2). shRNA targeting luciferase (shLuc) was used as a control. Interestingly, MEFs transduced with shLuc exhibited significantly increased basal levels of PLSCR2 compared to untreated cells (Figure S2C in Supplementary Material), which were efficiently depleted by shPLSCR2 (Figure 2A). Knockdown of PLSCR2 in MEFs significantly enhanced IFN-I-induced ISGs, including *Ifit1* and *Ifit3* (Figures 2B,C) and several other ISGs, compared to the shLuc control (Figures S3A–F in Supplementary Material). Similar phenomena were also observed in ML-1 cells (Figures S3G–K in Supplementary Material). To further investigate the significance of the enhanced ISG expression in the PLSCR2 knockdown, MEFs expressing shLuc or shPLSCR2 were infected with GFP-expressing SINV-GFP. PLSCR2 knockdown resulted in significantly reduced viral production at different MOIs compared to shLuc control, as revealed by decreased frequency of GFP^+^ cells (Figure 2D). We next pretreated the cells with a low dose of IFN-α overnight and found that IFN-mediated antiviral response was markedly enhanced in shPLSCR2 MEFs compared to shLuc control (Figure 2E). Similarly, an enhanced antiviral response against EMCV or VSV infection was also observed in MEFs expressing shPLSCR2 (Figures 2F,G).

To confirm the results seen in PLSCR2 knockdown MEFs, we generated ML-1 PLSCR2 knockout (PLSCR2KO) cells using the CRISPR-Cas9 system, as their basal levels of PLSCR2 are higher (Figure 2H). PLSCR2KO cells also showed increased ISG induction in response to IFN-I compared with the WT control (Figures 2I,J). To examine whether the enhanced IFN response in PLSCR2KO cells was intrinsic to the loss of PLSCR2, we restored PLSCR2 and found that the induction of ISGs was markedly decreased after re-expression of PLSCR2 compared to the EV control (Figures 2K,L). Likewise, PLSCR2KO cells also showed enhanced antiviral activity with attenuated replication of SINV-GFP (Figure 2M), and reduced viral titers in response to EMCV or VSV infection (Figures 2N,O). The elevated antiviral response in PLSCR2KO cells was not due to alteration of machinery for IFN-I production as the expression of pan-IFN-α following viral infection was comparable between WT and PLSCR2KO ML-1 cells (Figure S3L in Supplementary Material). Moreover, pretreatment with IFN-I further augmented the antiviral activity of PLSCR2KO cells (Figures 2M–O). In addition to RNA viruses, PLSCR2 appeared to exert a similar effect on innate immunity upon DNA virus infection as attenuated replication of DNA viruses, including vaccinia virus (VV) and ADV was also observed in PLSCR2KO ML-1 compared with WT ML-1 cells before and after IFN-I stimulation (Figures S4A,B in Supplementary Material). Together, these loss- and gain-of-function assays confirm that PLSCR2 negatively regulates IFN-I-mediated ISG induction and antiviral responses.

### PLSCR2KO Cells Exhibit Increased Basal and IFN-Induced Gene Expression

**Figure 3:**
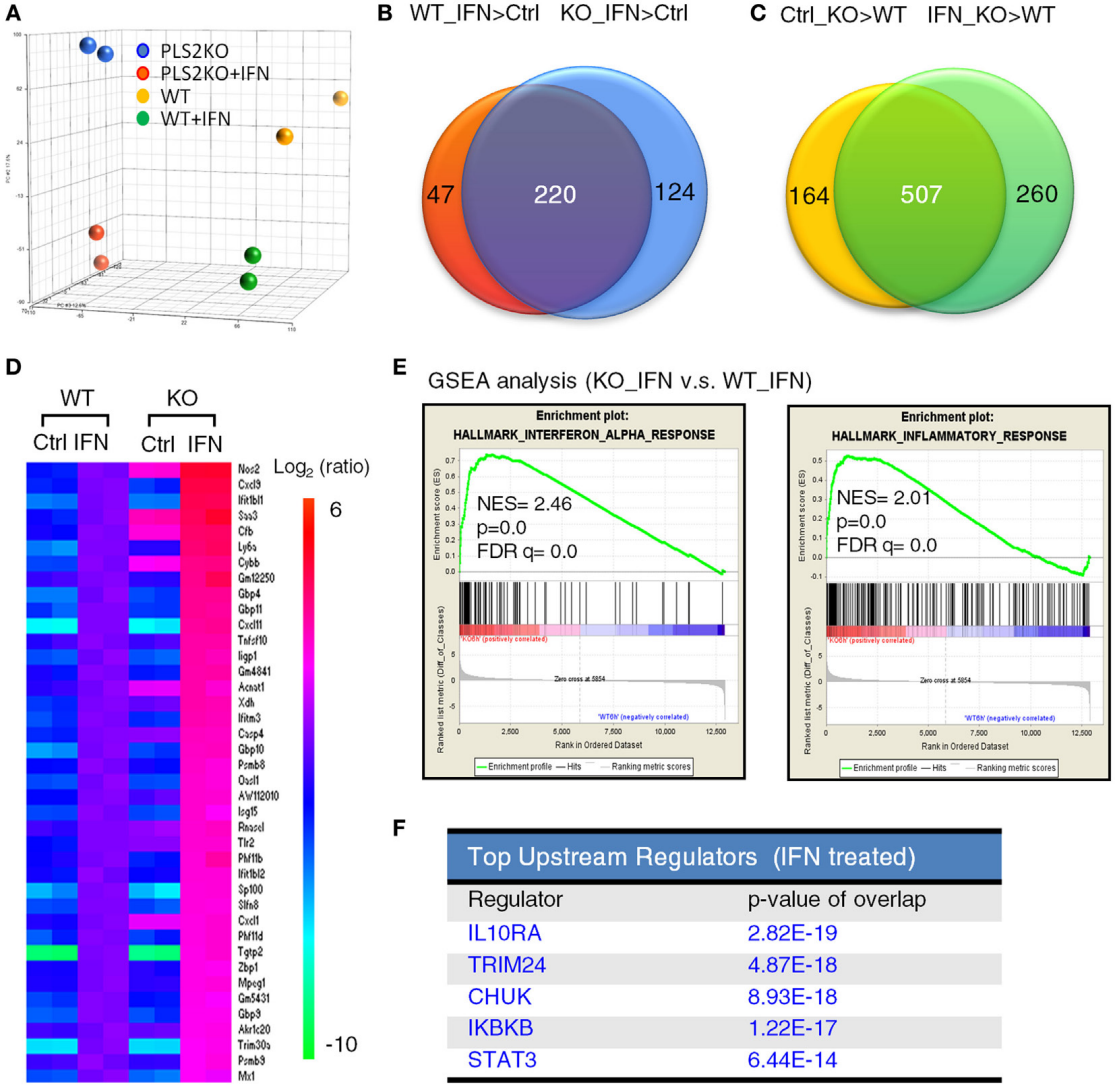
Phospholipid scramblase 2 (PLSCR2) deficiency enhances type I interferon (IFN-I)-induced antiviral and inflammatory gene signature. **(A)** Principal component analysis based on the gene expression profiles of WT and PLS2KO ML1 cells with or without IFN-α4 treatment for 6 h. **(B,C)** Venn diagram of shared upregulated genes (at least 2.0-fold changes and *p* < 0.05) in panel **(B)** WT (red) and PLS2KO (blue) cells following IFN-α4 6 h treatment vs. no treatment (ctrl), or in panel **(C)** no treatment (yellow) and IFN-α4 6 h treatment (green) in PLS2KO and WT cells. **(D)** Heatmap analysis of the upregulated IFN-stimulated genes (ISGs) in WT and PLS2KO cells. **(E)** Gene set enrichment analysis (GSEA) for IFN-α and inflammatory response genes. Abbreviations: NES, normalized enrichment score; FDR, false discovery rate. **(F)** Top upstream regulators analysis by ingenuity pathway analysis for IFN-I-treated WT vs. PLS2KO ML1 cells.

To better understand the regulatory roles of PLSCR2 on IFN-I response, we performed expression profiling on WT and PLSCR2KO ML-1 cells before and after IFN-I treatment using microarray analysis. Principal component analysis revealed that the expression profiles of the duplicated samples were very consistent (Figure 3A). While the genes induced by IFN-I treatment in PLSCR2KO cells highly overlapped with those in WT control (220 genes, 64%), the numbers of IFN-I-induced genes were increased in the absence of PLSCR2 (Figure 3B). Moreover, following IFN-I treatment in both PLSCR2 and WT control, there were 767 genes that were highly expressed in the absence of PLSCR2 compared to the WT control (IFN_KO > WT), of which 507 genes (75%) overlapped with the genes that were also higher in PLSCR2KO cells (Ctrl_KO > WT) without IFN-I treatment (Figure 3C), suggesting that PLSCR2 has an intrinsic property to suppress gene expression even without stimulation. Furthermore, heat map analysis confirmed that the expression of several ISGs were increased in PLSCR2KO cells compared to WT control following IFN stimulation, in which a selection of ISGs was verified by RT-QPCR (Figure 3D; Figures S5A–F in Supplementary Material). Moreover, basal levels of several ISGs, such as Nos2 and Tlr2, were also significantly higher (Figures S5E,F in Supplementary Material). Gene set enrichment analysis revealed that IFN-α and inflammatory response gene sets were highly enriched in PLSCR2KO cells compared with WT control following IFN-I stimulation (Figure 3E). Ingenuity pathway analysis also indicated that STAT3 was one of the upstream regulators of this pathway (Figure 3F), linking the signaling pathways of PLSCR2 to STAT3. Together, these analyses suggest that PLSCR2 is capable of exerting negative effects on IFN-I responses.

### The Suppressive Effects of PLSCR2 Are STAT3 Dependent

**Figure 4:**
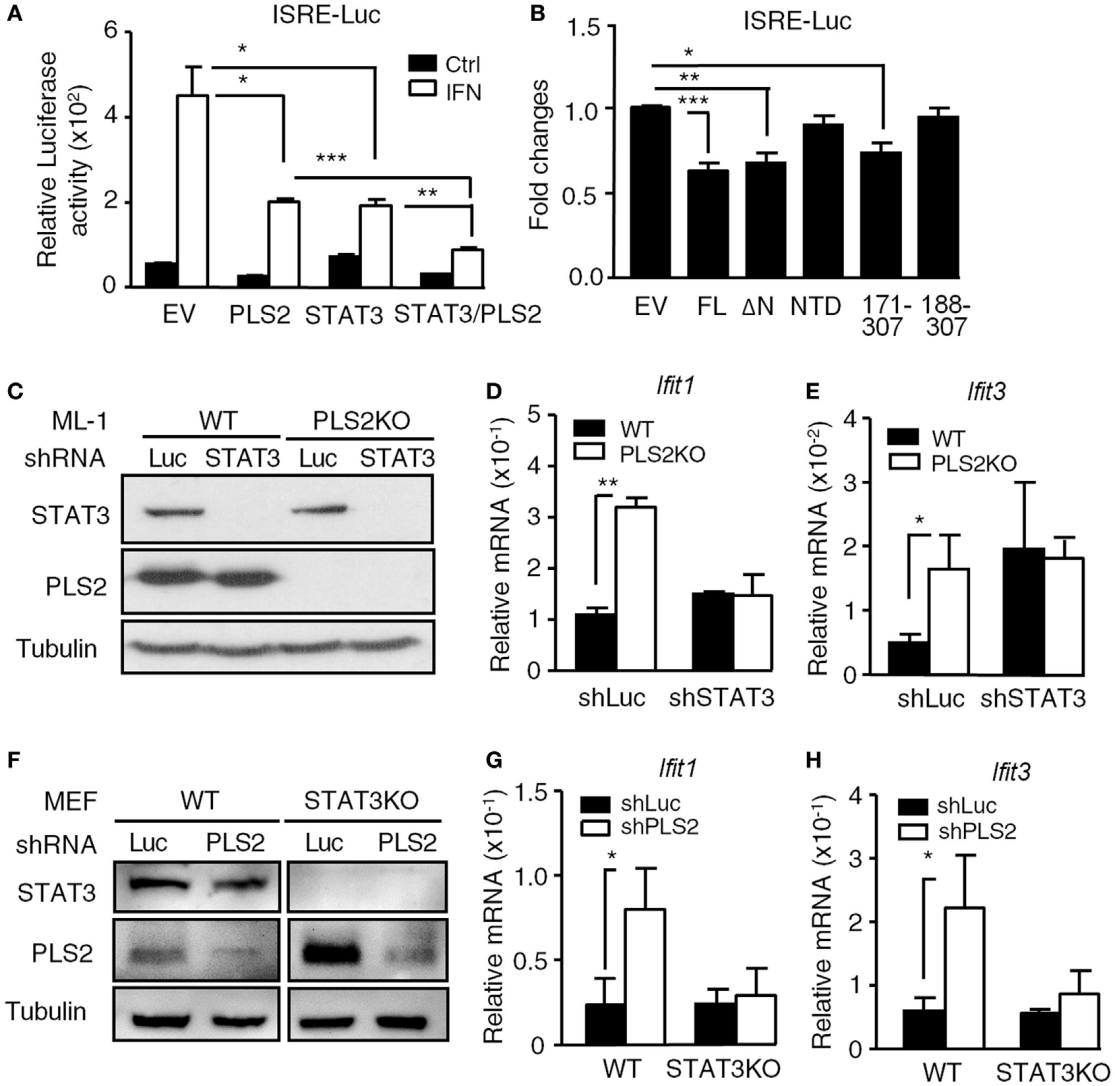
The suppressive effects of phospholipid scramblase 2 (PLSCR2) are STAT3 dependent. **(A)** ISRE-luc reporter plasmid was cotransfected with empty vector (EV) or vector expressing myc-PLSCR2 (PLS2) and/or HA-STAT3 and pEGFP-N1 in HEK293T cells for 24 h, followed by treatment with human IFN-α2 for 8 h. Total cell lysates were subjected to luciferase activity assay. Relative luciferase activity was normalized to the GFP^+^ percentage (*N* = 3). **(B)** Same as in panel **(A)**, except ISRE-luc was cotransfected with full length (FL) or different truncated mutants of PLSCR2 and pEGFP-N1 in HEK293T cells. Fold changes were normalized to EV control (*N* = 7). **(C)** WT and PLS2KO ML-1 cells were stably transduced with lentivirus expressing shLuc or shSTAT3. Total cell lysates were subjected to immunoblotting with antibodies against STAT3, PLSCR2, and tubulin. Blots are representative of two independent experiments. **(D,E)** WT or PLS2KO ML-1 cells stably transduced with shLuc or shSTAT3 were stimulated with mouse IFN-α4 for 6 h. Total RNA was subjected to RT-QPCR using primers to *Ifit1* **(D)**, *Ifit3* **(E)**, and *Rpl7*. Relative mRNA was normalized to *Rpl7* (*N* = 3). **(F)** WT and STAT3KO mouse embryonic fibroblasts (MEFs) were stably transduced with lentivirus expressing shLuc or shPLS2. Total cell lysates were subjected to immunoblotting as in panel **(C)**. **(G,H)** Same as in panels **(D,E)**, except WT or STAT3KO MEFs stably transduced with shLuc or shPLS2 were used (*N* = 3). Data are shown as mean ± SD, *p < 0.05, **p < 0.01, and ***p < 0.001.

Since PLSCR2 interacts with STAT3, we next examined whether the suppressive effects of PLSCR2 are dependent on STAT3. Overexpression of PLSCR2 or STAT3 alone in HEK293T cells suppressed ISRE-driven reporter activity in response to IFN-I, which was further aggravated by the combined expression of PLSCR2 and STAT3 (Figure 4A). We next evaluated the functional domains required for the suppressive activity of PLSCR2 using different truncation mutants. While full-length PLSCR2 (FL), PLSCR2_109–307_ (ΔN), and PLSCR2_171–307_ significantly suppressed ISRE reporter activity compared to the EV control, PLSCR2 NTD and PLSCR2_188–307_ did not (Figure 4B). Interestingly, the suppressive activity of PLSCR2 coincided with the ability of these mutants to interact with STAT3 (Figure 1B). Since PLSCR2_171–188_ contains the palmitoylation domain, these results also suggest that this domain is indispensable for the interaction with the STAT3 NTD and for its suppressive activities.

We next investigated the inter-dependency of STAT3 and PLSCR2 on the regulation of IFN-I response. ML-1 WT or PLSCR2KO cells were stably transduced with lentivirus carrying shRNA targeting luciferase or STAT3, followed by stimulation with IFN-I. shSTAT3 efficiently ablated STAT3 expression in both WT and PLSCR2KO ML-1 cells (Figure 4C). While PLSCR2KO cells exhibited enhanced IFN-I-induced expression of *Ifit1* and *Ifit3*, knockdown of STAT3 abrogated the effect (Figures 4D,E). To confirm these findings, we also performed a reversed experiment by knocking down PLSCR2 in WT or STAT3KO MEFs. As shown in Figure 4F, the expression of PLSCR2 was efficiently ablated by shPLSCR2 in both WT and STAT3KO MEFs (Figure 4F). However, when PLSCR2 was depleted, the enhancement of IFN response in STAT3KO MEFs was also impeded (Figures 4G,H). These results suggest that there is an interdependent requirement for STAT3 and PLSCR2 during the negative regulation of IFN-I response.

### Palmitoylation of PLSCR2 Is Required for Its Interaction With STAT3 and for Its Suppressive Activities

**Figure 5:**
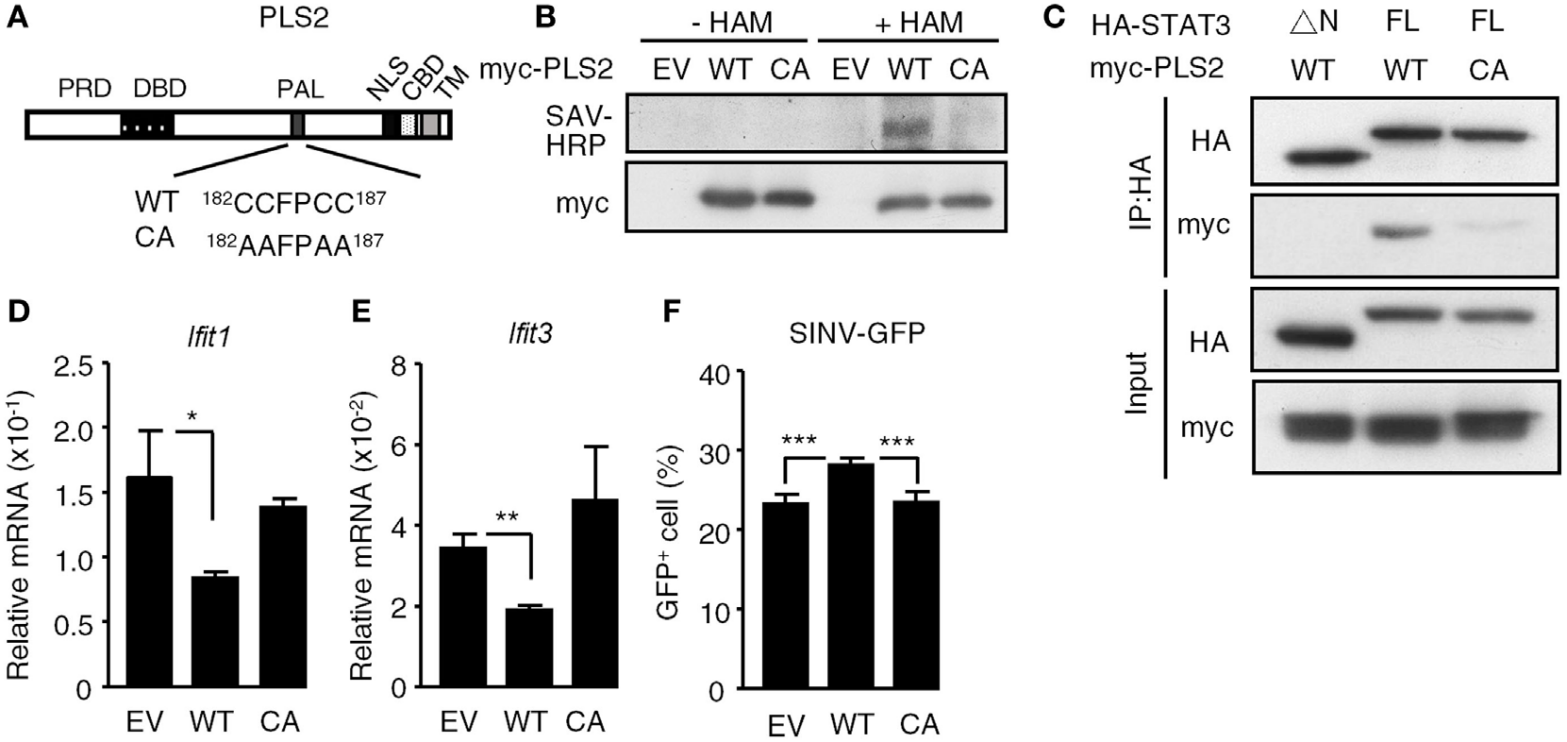
Palmitoylation of phospholipid scramblase 2 (PLSCR2) is required for interaction with STAT3 and for its suppressive effects. **(A)** Schematic diagram of WT and CA mutant of PLSCR2. Four conserved cysteine residues (CCFPCC) in the palmitoylation motif were changed to alanine residues. **(B)** HEK293T cells were transfected with empty vector (EV), myc-tagged WT, or CA mutant of PLSCR2 for 48 h and were subjected to palmitoylation assay as described in Section “Materials and Methods.” Total cell lysates were subjected to blotting with SAV-horseradish peroxidase or anti-myc antibody. Blots are representative of two independent experiments. **(C)** HEK293T cells were cotransfected with HA-tagged ΔN or full length (FL) STAT3 and myc-tagged WT, or CA mutant of PLSCR2. Co-IP assays were performed with anti-HA antibody followed by immunoblotting with antibodies against HA or myc. Immunoblotting of total cell lysates was used as input control. Blots are representative of two independent experiments. **(D,E)** PLS2KO ML-1 cells were transfected with EV, WT, or the CA mutant of PLSCR2 for 48 h. The transfected cells were treated with mouse IFN-α4 for 6 h. Total RNA of the treated cells was subjected to RT-QPCR using primers to *Ifit1* **(D)**, *Ifit3* **(E)**, and *Rpl7*. Relative mRNA was normalized to *Rpl7* (*N* = 3). **(F)** Same as in panels **(D,E)** except the transfected cells were infected with Sindbis virus (SINV)-GFP at an MOI of 10 for 20 h, followed by flow cytometric analysis for GFP^+^ cells (*N* = 4). Data are shown as mean ± SD, *p < 0.05, **p < 0.01, and ***p < 0.001.

Since the palmitoylation domain of PLSCR2 is required for the functional interaction with STAT3, we next determined whether palmitoylation of PLSCR2 controls its biological activities. We generated a palmitoylation-deficient mutant based on a report (24) by changing four conserved cysteine residues into alanine (CA mutant) and examined the resulting palmitoylation activity (Figure 5A). Indeed, WT PLSCR2 was labeled with biotinylated substrate following treatment of cells with HAM, whereas the CA mutant was not (Figure 5B). In addition, the interaction between PLSCR2 and STAT3 was greatly diminished by the CA mutation (Figure 5C). The suppressive effect of PLSCR2 on IFN-I-induced ISGs, such as *Ifit1* and *Ifit3*, was also blunted by the CA mutation of PLSCR2 (Figures 5D,E) as was the production of SINV-GFP (Figure 5F). These results suggest that palmitoylation of PLSCR2 is required for its association with STAT3 and for the negative regulation of IFN response.

### PLSCR2 Suppresses the Recruitment of ISGF3 to Promoters of ISGs

**Figure 6:**
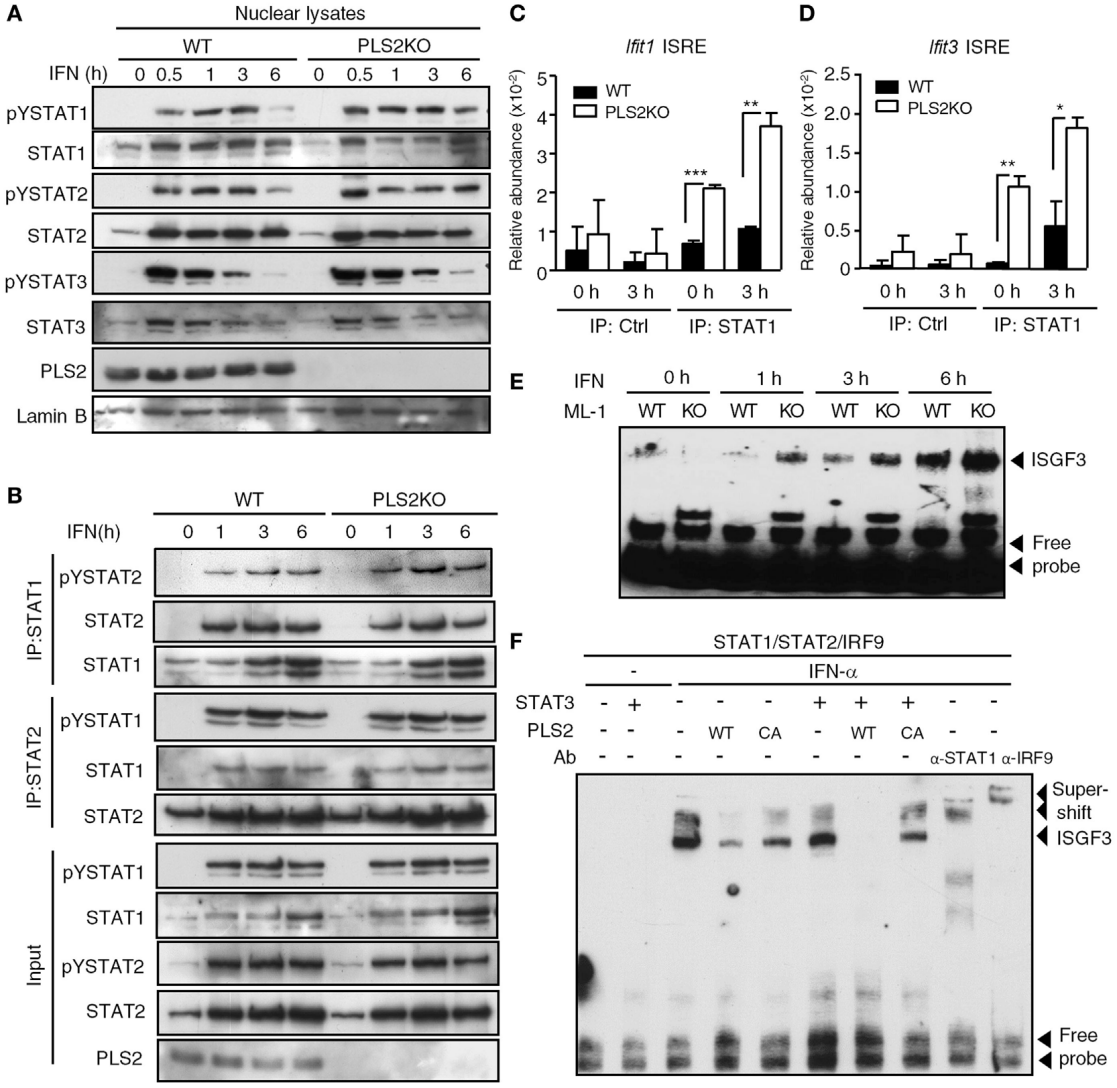
Phospholipid scramblase 2 (PLSCR2) suppresses the recruitment of ISGF3 to IFN-stimulated gene (ISG) promoters. **(A)** WT and PLS2KO ML-1 cells were treated with IFN-α4 for the indicated times. Nuclear extracts were subjected to immunoblotting with antibodies against pYSTAT1, STAT1, pYSTAT2, STAT2, pYSTAT3, STAT3, PLSCR2, or lamin B. Blots are representative of three independent experiments. **(B)** Same as in panel **(A)**, except nuclear extracts were subjected to co-IP using antibodies against STAT1 or STAT2, followed by immunoblotting with antibody to pYSTAT2 or pYSTAT1, STAT2, and STAT1. Total cell extracts were subjected to immunoblotting using the indicated antibodies for input control. Blots are representative of two independent experiments. **(C,D)** WT and PLS2KO ML-1 cells were treated with IFN-α4 for 3 h, followed by chromatin immunoprecipitation with antibody to control Ig (Ctrl) or STAT1 and then subjected to Q-PCR using primers to ISRE of the promoter of *Ifit1* **(C)** or *Ifit3* **(D)**. Relative abundance was normalized to input (*N* = 3). **(E)** WT and PLS2KO ML-1 cells were stimulated with or without IFN-α4 for the indicated times. Nuclear extracts of the treated cells were subjected to electrophoretic mobility shift assay (EMSA) as described in Section “Materials and Methods.” Blots are representative of two independent experiments. **(F)** HEK293T cells were transfected individually with empty vector (EV), HA-tagged STAT3, myc-tagged WT or CA mutant of PLS2 or cotransfected with human STAT1, STAT2, and IRF9 for 48 h and then treated with or without IFN-α2 for 60 m. The nuclear lysates were added together *in vitro* as indicated before EMSA. For the supershift assay, anti-STAT1 or anti-IRF9 antibodies were added to the mixture. Blots are representative of two independent experiments. Data are shown as mean ± SD, **p < 0.01, and ***p < 0.001.

We next investigated the mechanisms by which PLSCR2 suppresses IFN-I response. PLSCR2 deficiency did not exert any notable changes on IFN-dependent tyrosine phosphorylation of STAT1, STAT2, or STAT3 (Figure S6A in Supplementary Material). Nor did loss of PLSCR2 affect the nuclear translocation of the activated STAT proteins (Figure 6A). A similar result was also observed in MEFs expressing shPLSCR2 compared with the shLuc control (Figure S6B in Supplementary Material). We next examined the effect of PLSCR2 on ISGF3 assembly through co-IP experiments. The presence of exogenous PLSCR2 did not alter IFN-induced interaction of STAT1, STAT2, or IRF9, while it did slightly reduce the association of unphosphorylated STAT1 and STAT2 (Figure S6C in Supplementary Material). Moreover, IFN-I-stimulated interaction of endogenous STAT1 and STAT2 was also comparable between WT and PLSCR2KO ML-1 cells (Figure 6B). However, exogenous or endogenous PLSCR2 co-immunoprecipitated by the anti-STAT2 antibody was much abundant than that by the anti-STAT1 antibody (Figures S6C,D in Supplementary Material). This is probably due to the fact that PLSCR2 only interacts with STAT2, and not with STAT1 or IRF9 (Figure 1D). Together, these results suggest that the suppressive effect of PLSCR2 is not mediated by blocking the activation, nuclear translocation, or assembly of ISGF3 components.

We next examined the effect of PLSCR2 on promoter occupancy by ISGF3 complex using a ChIP assay. Recruitment of ISGF3 to the ISREs of *Ifit1* and *Ifit3* was found to be enhanced in PLSCR2KO cells compared with the WT control before and after IFN-I treatment (Figures 6C,D), suggesting that PLSCR2 may suppress the binding of ISGF3 to ISRE. To further dissect the detailed underlying mechanisms, we performed an EMSA to assess the effect of PLSCR2 on the DNA binding of ISGF3 *in vitro*. Indeed, increased binding of ISGF3 to ISRE was observed in PLSCR2KO cell extracts following IFN treatment compared with WT control (Figure 6E), suggesting that PLSCR2 may prevent ISGF3 from binding to ISRE-containing DNA. To clarify the contributions of STAT3 and PLSCR2 to this blocking of DNA binding by ISGF3, WT or CA mutant PLSCR2-transfected cell extracts were incubated with preformed ISGF3 in the presence or absence of STAT3. IFN-I treatment resulted in rapid induction of DNA binding by ISGF3, which was supershifted by anti-STAT1 or anti-IRF9 antibody (Figure 6F). The binding of ISGF3 to ISRE was attenuated by the presence of WT PLSCR2, and, to a lesser extent, by the CA mutant. Moreover, STAT3 and WT PLSCR2 augmented the suppressive effect on DNA binding by ISGF3, which was abolished by the CA mutant (Figure 6F). These results suggest that the PLSCR2–STAT3 axis exerts a negative effect on IFN-I response by suppressing ISGF3 binding to DNA.

### The Suppressive Effect of PLSCR2 Is Also STAT2-Dependent

**Figure 7:**
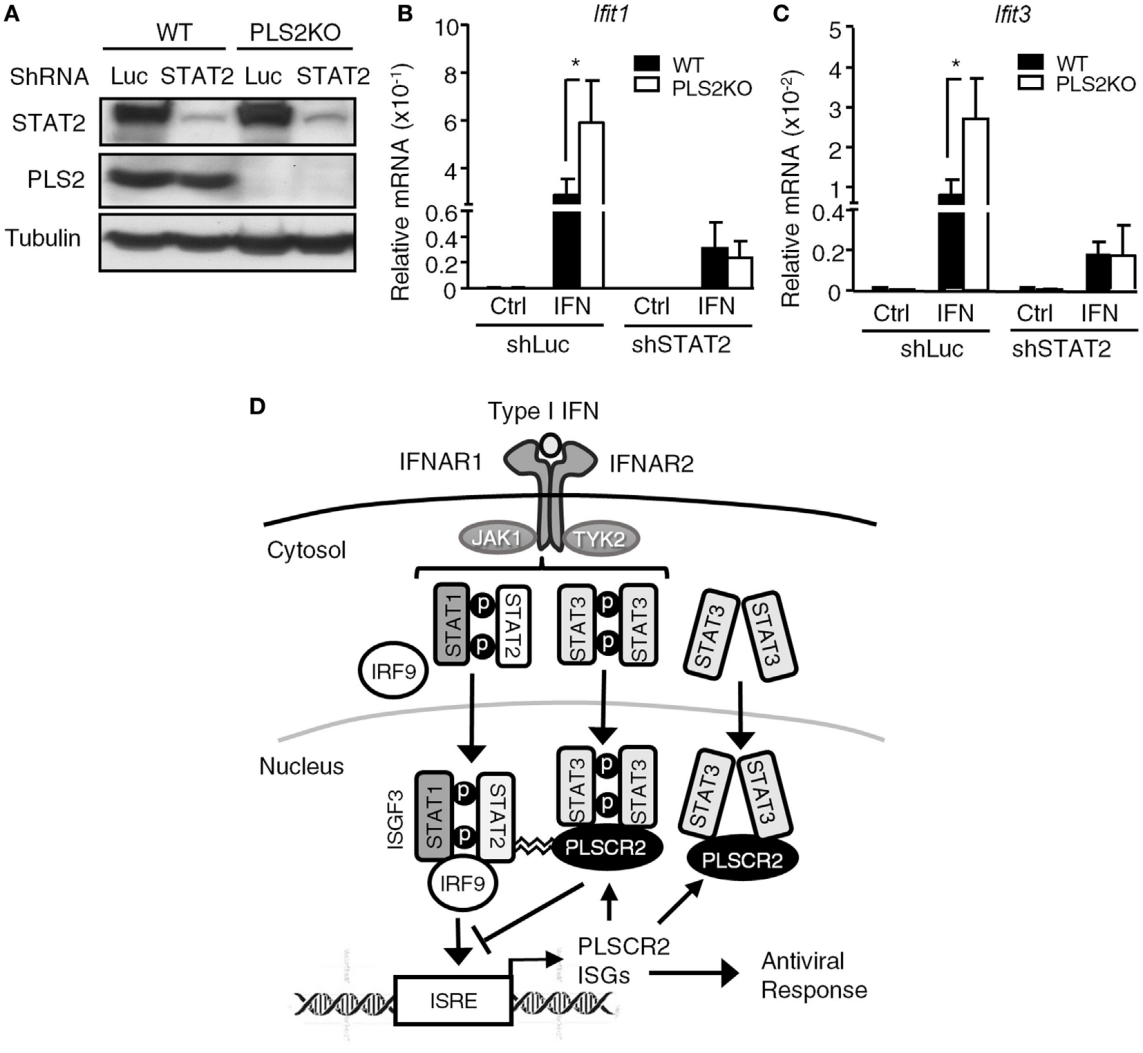
The suppressive effect of phospholipid scramblase 2 (PLSCR2) is also STAT2-dependent. **(A)** WT and PLS2KO ML-1 cells were stably transduced with shLuc or shSTAT2. Total cell lysates were subjected to immunoblotting with antibodies against STAT2, PLSCR2, or tubulin. **(B,C)** WT or PLS2KO ML-1 cells stably transduced with shLuc or shSTAT2 were stimulated with or without IFN-α4 for 6 h. Total RNA was subjected to RT-QPCR using primers to *Ifit1* **(B)**, *Ifit3* **(C)**, and *Rpl7*. Relative mRNA was normalized to *Rpl7* (*N* = 3). Data are shown as mean ± SD. *p < 0.05. **(D)** A model of fine tuning of type I interferon (IFN-I) response by the STAT3–PLSCR2 axis. The binding of IFN-I to IFN receptor activates STAT1, STAT2, and STAT3. Activated STAT1 and STAT2 form ISGF3 complex with IRF9, translocate into the nucleus, bind the promoters, and transactivate downstream IFN-stimulated genes (ISGs) resulting in an antiviral response. One of the ISGs is PLSCR2, which interacts with the N-terminal domain (NTD) of phosphorylated or unphosphorylated STAT3 through its palmitoylation motif. The STAT3–PLSCR2 complex impedes the recruitment of ISGF3 to the promoters, probably in a STAT2-dependent manner, thus suppressing IFN-induced ISGs and the antiviral response.

While PLSCR2 and STAT3 were capable of inhibiting the promoter occupancy of ISGF3, it remains unclear how they acquired this ability. Since PLSCR2 also physically interacts with STAT2 (Figure 1D; Figures S6C,D in Supplementary Material), a component of ISGF3, we reasoned that PLSCR2 might exert this inhibitory effect by direct binding to ISGF3 through STAT2 and impeding DNA-binding and transcriptional activities. To test this possibility, we knocked down STAT2 in WT or PLSCR2KO cells using shRNA. The endogenous STAT2 was significantly depleted by shSTAT2 in both cell types when compared with the shLuc control (Figure 7A). Moreover, compared with controls, IFN-I-induced expression of *Ifit1* and *Ifit3* was greatly enhanced in the absence of PLSCR2, while shSTAT2 severely impaired IFN-I-induced expression of both of these ISGs in both cell types (Figures 7B,C). However, a low, but significant, level of expression of these ISGs compared to the untreated control was still observed; however, PLSCR2KO-mediated enhancement of ISG expression was blunted by STAT2 deficiency. These results suggest that the suppressive effect of PLSCR2–STAT3 on IFN-I response is also STAT2 dependent.

## Discussion

While PLSCR2 was one of the first mouse PLSCR family members identified, little is known about its biological role. In this study, we highlight a novel regulatory role of the PLSCR2–STAT3 axis in suppressing IFN-I response. We demonstrate that: (1) PLSCR2 constitutively interacts with the STAT3 NTD through its palmitoylation domain, (2) PLSCR2 is induced by IFN-I and is predominantly localized to the nucleus, (3) PLSCR2 suppresses IFN-I-induced antiviral response in a STAT3-dependent manner, and (4) PLSCR2 palmitoylation is indispensable for binding to the STAT3 NTD and inhibition of the recruitment of ISGF3 to promoters. Therefore, this study extends our previous findings (4) and characterizes the role of this newly identified molecule in regulating IFN-I response.

STAT3 possess the ability to translocate to the nucleus independent of its activation status, although ligand-induced tyrosine phosphorylation of STAT3 increases nuclear accumulation (43). Overexpression of exogenous STAT3 results in its constant presence in the nucleus, particularly in nuclear bodies (44). Here, we found that PLSCR2 is also mainly located to the nucleus and is constitutively associated with nuclear STAT3. This may explain the finding that IFN treatment did not affect the interactions between exogenous STAT3 and PLSCR2, but did increase endogenous STAT3/PLSCR2 complex formation.

Palmitoylation of PLSCR family members is critical for their subcellular distribution (24). All PLSCR family members have a tendency for nuclear localization, which highly correlates with their palmitoylation state (18–20). However, our results suggest that PLSCR2 predominantly resides in the nucleus regardless of its palmitoylation status, which is consistent with a previous report (21). While palmitoylation mainly controls reversible lipidation, it has also been reported to contribute to subcellular trafficking, protein–protein interactions, hydrophobicity, and stability (16, 17, 45). Although palmitoylation is not a common posttranslational modification for transcription factors and nuclear proteins, increasing evidence suggests that palmitoylation in the nucleus may influence gene transcription, chromatin structure, and epigenetic events (46–48). In this study, palmitoylation-deficient PLSCR2 was found to exhibit a reduced ability to interact with STAT3 and to suppress IFN-I response. It is possible that palmitoylation of PLSCR2 may increase its affinity for STAT3 as well as its ability to inhibit IFN-I response. Thus, the next step will be to determine if PLSCR2 palmitoylation results in the sequestration of IFN-I-induced ISGF3 complex by membrane targeting.

Like PLSCR2, PLSCR1 is also induced by IFN-I (27); however, PLSCR1 has been shown to be a positive regulator of the antiviral response against EMCV, VSV, HBV, and HIV (27, 28, 49). Furthermore, PLSCR1KO MEFs display increased viral titers compared with WT control (27). Plasmacytoid dendritic cells from PLSCR1KO mice also produce less type I IFN upon CpG stimulation or HSV infection (26). Mechanistically, PLSCR1 regulates TLR9 trafficking to the endosomal compartment and is required for IFN-I-mediated protection against staphylococcal α-toxin. Nevertheless, the molecular mechanisms underlying the effects of PLSCR1 on the antiviral response remains elusive. Structurally, all members of the PLSCR family share a similar transcription factor-like domain, which contains a PRD, DBD, and NLS. However, it is noteworthy that PLSCR1 and PLSCR2 may play opposite roles in cell activation downstream of Lyn signaling (50, 51). Here, we also show that PLSCR2 antagonizes IFN-I-mediated antiviral response in a STAT3-dependent manner.

Based on this study, we propose a model to illustrate the potential mechanism for the suppressive activity of the STAT3–PLSCR2 axis on IFN-I response (Figure 7D). In this model, the engagement of IFN-I to IFN receptor activates the promoter binding by ISGF3 complex and the transactivation of downstream ISGs, thus mediating antiviral response. One such ISG is PLSCR2, which through its palmitoylation motif interacts with phosphorylated or unphosphorylated STAT3. The STAT3–PLSCR2 complex impedes the recruitment of ISGF3 to the promoters, probably in a STAT2-dependent manner, thus suppressing IFN-induced ISGs and antiviral response. Interestingly, yellow fever virus employs a similar strategy to evade IFN-I response, through an interaction of its NS5 protein with tyrosine phosphorylated STAT1-bound STAT2 and the prevention of engagement of ISREs by ISGF3 (52). While the detailed mechanisms of the action remain to be determined, no apparent sequence similarity was found between NS5 and PLSCR2 (Figure S6 in Supplementary Material), suggesting that the suppression mechanisms of these two molecules are most likely distinct. Many host factors are reported to negatively regulate the production of IFN-I through targeting innate sensors, such as RIG-I, MAVS, and MDA5 and transcription factors IRF3/7 or IFN-I signaling through targeting signal mediators, such as IFNAR1/2, STAT1, and STAT2 (53). Recently, TRIM29, a E3 ubiquitin ligase that is highly expressed in the lung alveolar macrophages, is also found to be critical for controlling RNA and DNA virus-triggered airway inflammation by targeting NEMO (IKKγ) and STING, respectively, to attenuate the production of IFN-I and proinflammatory cytokines (54, 55). Nevertheless, the mode of action of TRIM29 is completely different from that of PLSCR2, as the latter does not alter the production of IFN-I during viral infection. Due to low basal levels of PLSCR2 in MEFs and some lymphoid tissues such as bone marrow cells, it is reasonable to assume that PLSCR2 may be part of a negative feedback loop to control and restrict the extent and duration of the innate antiviral response under physiological conditions, such as is known to occur with the suppressors of cytokine signaling and USP18 (56).

STAT3 is versatile molecule involved in various cell functions including survival, apoptosis, proliferation, differentiation, and migration and is required for embryogenesis, neurogenesis, hematopoiesis, and other biological processes (57). Like other STAT family members, the function of STAT3 is controlled by a wide range of mechanisms including posttranslational modification, protein turnover, nuclear import, negative regulation, and cofactor binding (58). Although STAT3 has been shown to interact with different proteins to regulate its activity (59–61), PLSCR2 appears to be unique as it cooperates with STAT3 to achieve its suppressive effects. Accumulating evidence suggests that STAT3 not only negatively regulates innate immunity (62) but also attenuates adaptive immunity against tumors (63). Therefore, these findings may open a new avenue for studying the regulatory role of STAT3 and provide a novel therapeutic target to improve antiviral and antitumor responses.

## Data Availability

The microarray data from this publication have been deposited to the GEO database https://www.ncbi.nlm.nih.gov/geo/query/acc.cgi?acc=GSE110967 and the assigned accession number is GSE110967.

## Ethics Statement

All infection experiments were performed following the guidelines of biosafety level 2 approved by the Environmental Protection and Occupational Safety and Health Center at National Taiwan University.

## Author Contributions

M-HT designed and conducted experiments, analyzed data, and wrote the manuscript. C-KL conceived the idea, analyzed data, wrote the manuscript, and supervised the project.

## Conflict of Interest Statement

The authors declare that the research was conducted in the absence of any commercial or financial relationships that could be construed as a potential conflict of interest.
