# Supplementary material for: STAT3 Cooperates With Phospholipid Scramblase 2 to Suppress Type I Interferon Response
Source: Front Immunol. 2018 Aug 15;9:1886. doi: 10.3389/fimmu.2018.01886 (PMC6104169; doi:10.3389/fimmu.2018.01886)
Supplement: Supplementary file 1 [file Data_Sheet_1.PDF]

Figure S1

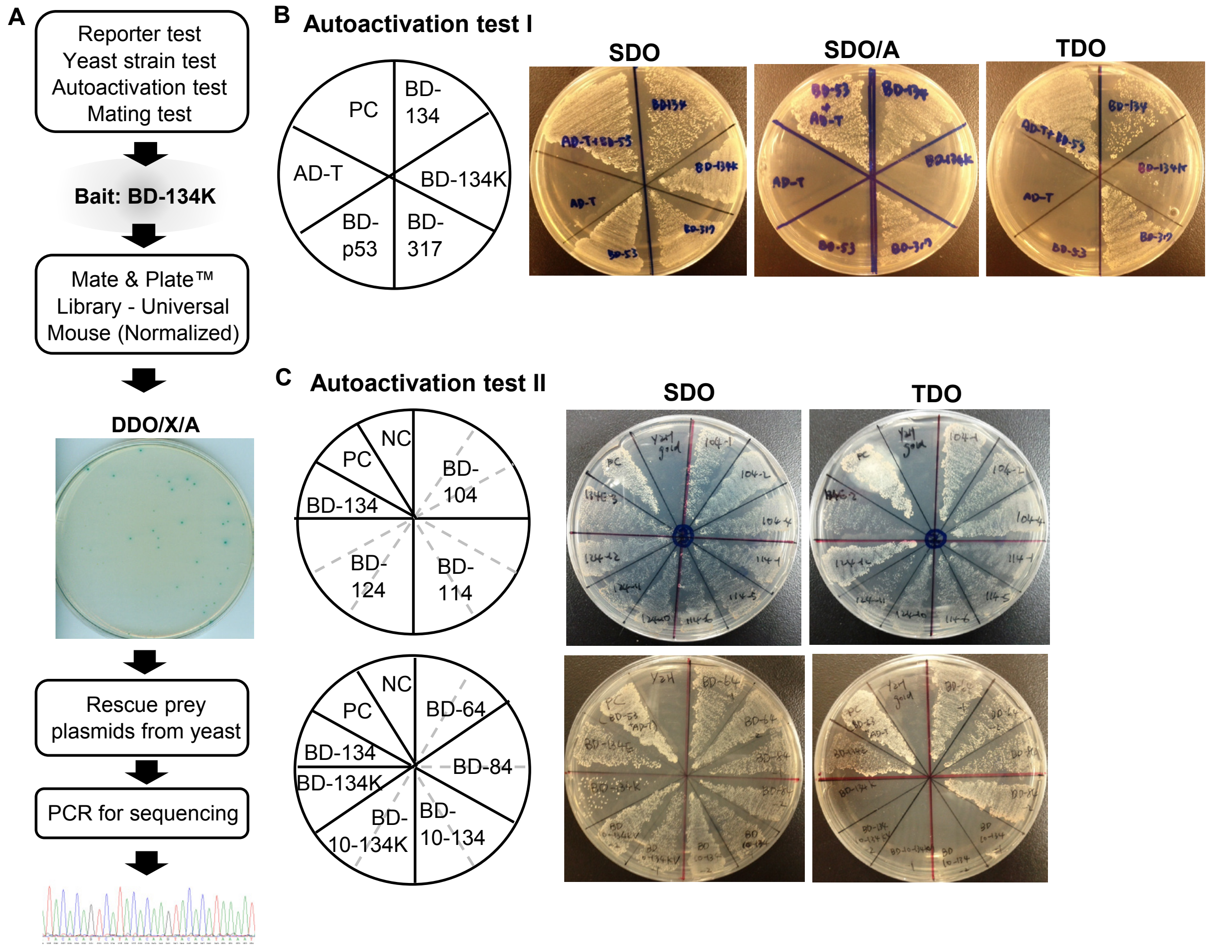

D

Identified STAT3 NTD interacting genes		
ACY1	LYPLA1	SRR
ARID1B	MED21	ST6GALNAC2
CCT3	METAP2	TIMP4
CCT4	NANP	TPP1
CCT6A	NDUFA9	TYROBP
CLTC	OSCP1	ULK2
CTSO	PAICS	USP9X
DGCR8	PANK1	WSB2
DLD	PCMT1	
F13A1	PDHX	
FNTA	PLA1A	
FOLH1	PLSCR2	
FUCA2	RANBP9	
GNB1	RCE1	
GNB4	RHOT1	
GNB5	RRM2	
HIBADH	SF3B1	
HOMEZ	SH3GLB1	
IIGP1	SLC1A1	
ISG20L2	SLC33A1	
IVNS1ABP	SLC43A3	
LDHA	SLC6A19	
LGALS3BP	SLC7A12	
LYN	SPINK12	

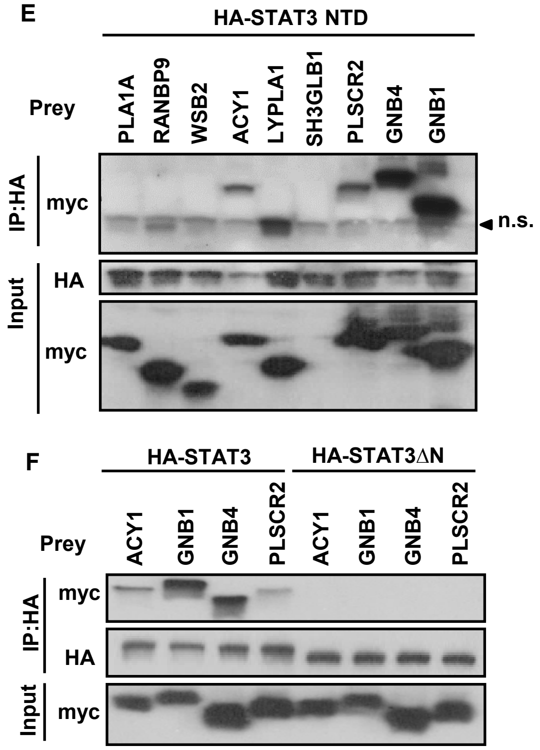

Figure S1. Identification of STAT3 NTD-interacting proteins using the yeast two-hybrid system. (A) A flowchart of the yeast two-hybrid system for the identification of the STAT3 NTD-interacting proteins. BD, GAL4 DNA-binding domain; DDO/X/A: Trp/Leu dropout+ X-alpha-gal+ Aureobasidin A (B) Bait plasmid BD-134, BD-317, or BD-134K was transformed into Y2Hgold strain yeast and then plated on selective plates. AD, GAL4 activation domain; SDO: Trp single dropout; TDO: Trp/His/Ade Triple dropout; SDO/A: Trp single dropout+ Aureobasidin A; PC (positive control): AD-T/BD-p53 cotransformant; NC (negative control): Y2Hgold strain without transformation. (C) Same as in (B), except bait plasmids BD-124, BD-114, BD-104, BD-84, BD-64, BD-10-134, and BD-10-134K were used. (D) STAT3 NTD-interacting genes following sequence analysis of the yeast two-hybrid system using BD-134K as bait. (E) The rescued prey plasmids from yeast were subcloned into mammalian expression vector pCMV-myc and co-transfected with HA-tagged STAT3 NTD into HEK293T cells, followed by co-IP with anti-HA antibody and immunoblotting with antibody to myc and HA, respectively. (F) Same as in (E), except the selected prey constructs were cotransfected with HA-STAT3 or $STAT3_{135\text{-}770}$ (ΔN).

Figure S2

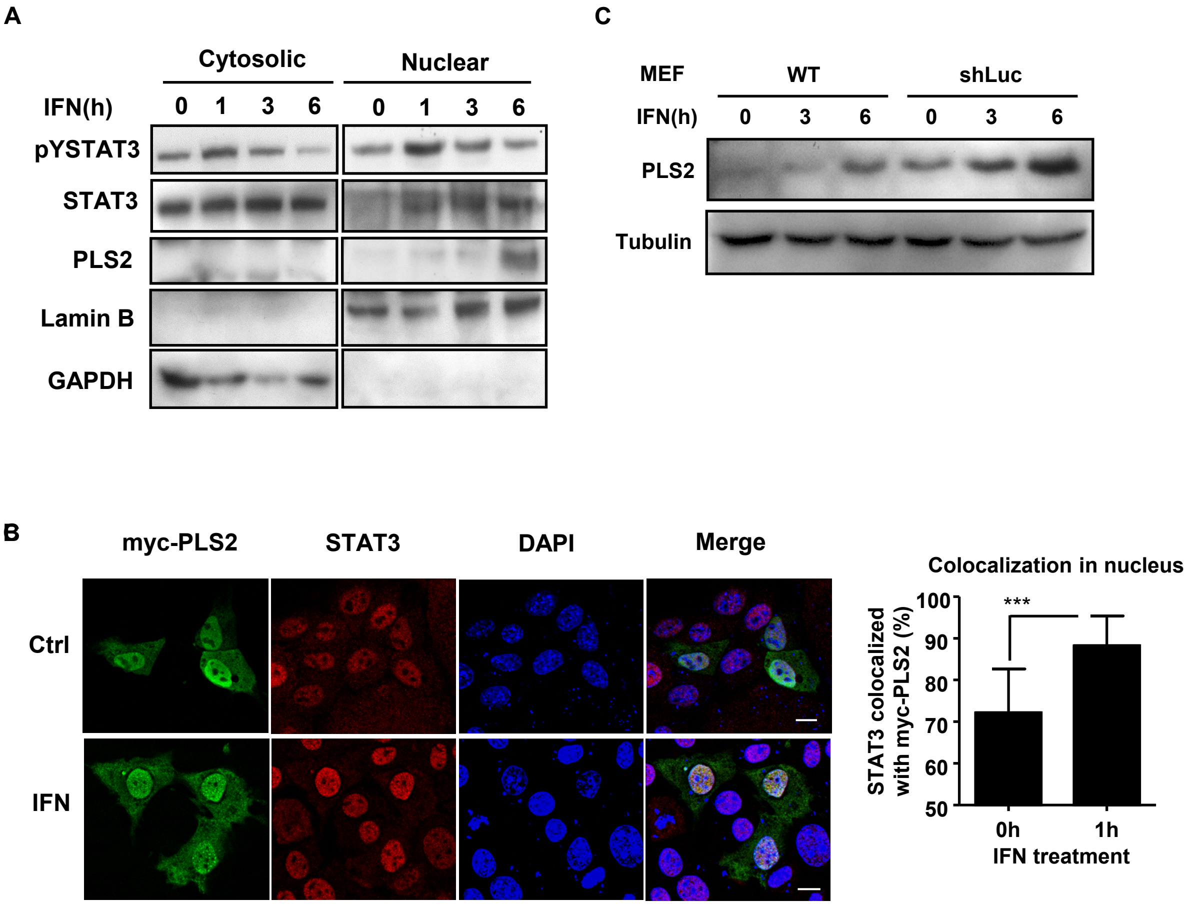

Figure S2. IFN-I-induced PLSCR2 predominantly locates to the nucleus. (A) WT MEFs were stimulated with or without mouse IFN-α4 (1000 U/ml) for the indicated times. Cytosolic and nuclear lysates were subjected to immunoblotting with antibodies against PLSCR2, pSTAT3, STAT3, lamin B, or GAPDH. (B) PLS2KO ML-1 cells were transfected with myc-PLSCR2 for 24 h and treated with or without mouse IFN-α4 for 1 h, fixed, and then stained with primary mouse antibody to myc and rabbit antibody to STAT3 and secondary antibodies to FITC-conjugated anti-mouse IgG and DyLight 594-conjugated anti-rabbit IgG, respectively, and DAPI. Samples were visualized by confocal microscopy. Quantification of myc-PLSCR2/STAT3 colocalization in the nuclear area are shown as mean ± SD. (C) WT MEFs and MEFs expressing luciferase (shLuc) shRNA were treated with or without mouse IFN-α4 (1000 U/ml) for the indicated times and then were subjected to immunoblotting with antibodies to PLSCR2 and tubulin. Scale bar = 10 μm. ***$p < 0.001$, n=15.

Figure S3

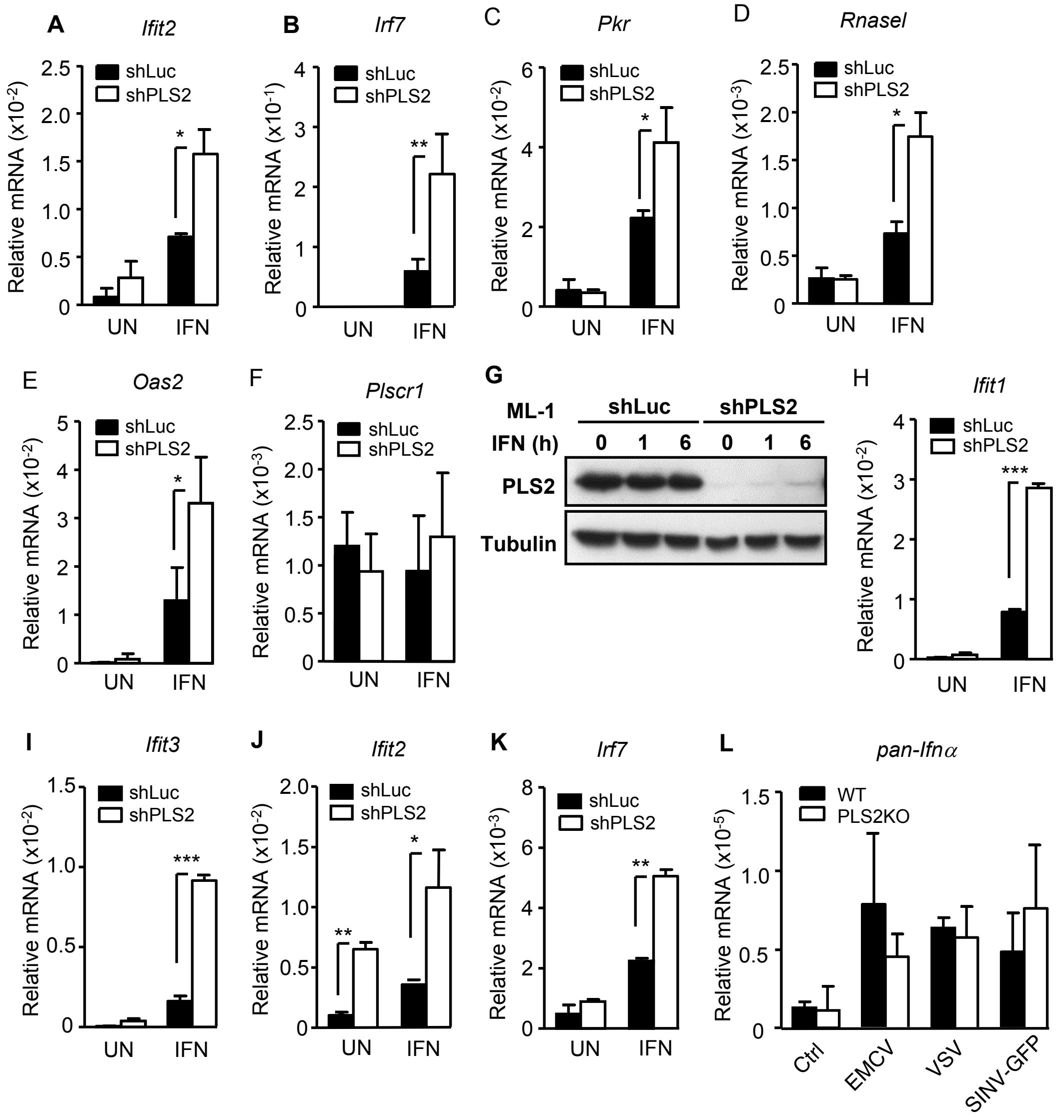

Figure S3. PLSCR2 knockdown enhances the IFN-induced ISG expression. WT MEFs expressing luciferase- (shLuc) or PLSCR2-specific (shPLS2) shRNA were treated with or without mouse IFN-α4 (1000 U/ml) for 6 h and RNA was subjected to RT-QPCR using primers to mouse *Ifit2* (A)*, Irf7* (B)*, Pkr* (C)*, Rnasel* (D), *Oas2* (E), *Plscr1* (F), and *Rpl7*. Relative mRNA was normalized to *Rpl7.* (G) ML-1 cells expressing luciferase- (shLuc) or PLSCR2-specific (shPLS2-1) shRNA were treated with mouse IFN-α4 (1000 U/ml) for the indicated times. Total cell lysates were subjected to immunoblotting with antibody against PLSCR2 or tubulin. The cells stimulated with IFN-α4 for 6 h were subjected to RT-QPCR using primers to mouse *Ifit1* (H), *Ifit3* (I), *Ifit2* (J), *Irf7* (K) and *Rpl7*. (L) WT or PLSCR2KO cells were infected with or without the indicated viruses for 6 h and were subjected to RT-QPCR using primers to pan-*Ifnα* and *Rpl7*. Relative mRNA was normalized to *Rpl7*. Data are shown as mean ± SD. *p < 0.05, **p < 0.01, and ***p < 0.001

Figure S4

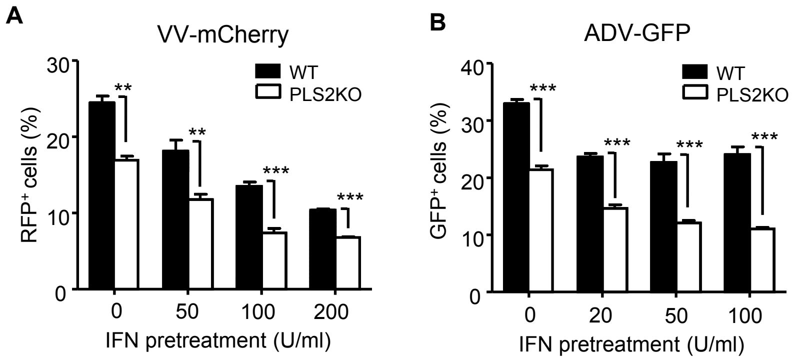

Figure S4. PLSCR2 deficiency enhances IFN-I-induced antiviral responses against DNA virus. (A,B) WT and PLS2KO ML-1 cells were treated with or without mouse IFN-α4 at indicated doses for 24 h and then infected with VV-mCherry (A) for 20 h or ADV-GFP (B) for 24 h at an MOI of 1 followed by flow cytometric analysis for RFP^+ or GFP^+ cells (N=6). Data are shown as mean ± SD. *$p < 0.05$, **$p < 0.01$, and ***$p < 0.001$

Figure S5

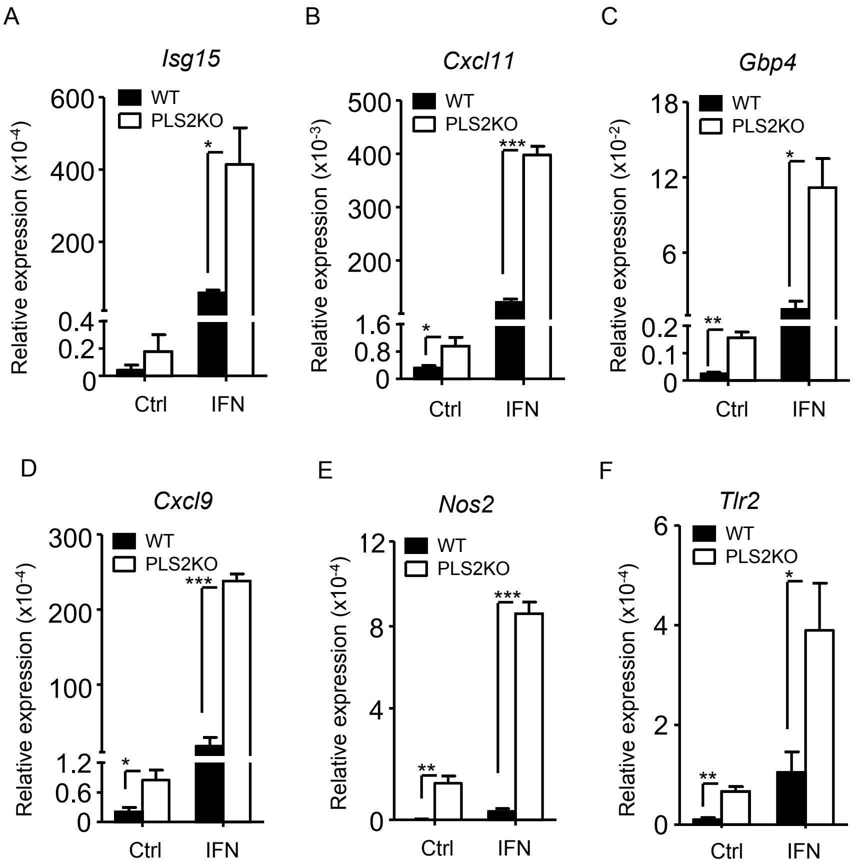

Figure S5. Confirmation of microarray data by RT-QPCR. WT and PLS2KO ML-1 cells were treated with or without mouse IFN-α4 for 6 h. Total RNA was subjected to RT-QPCR using primers to *Isg15* (A), *Cxcl11* (B), *Gbp4* (C), *Cxcl9* (D), *Nos2* (E), *Tlr2* (F), *and Rpl7*. Relative mRNA was normalized to *Rpl7* (N=2). Data are shown as mean ± SD. $*p < 0.05$, $**p < 0.01$, and $***p < 0.001$

Figure S6

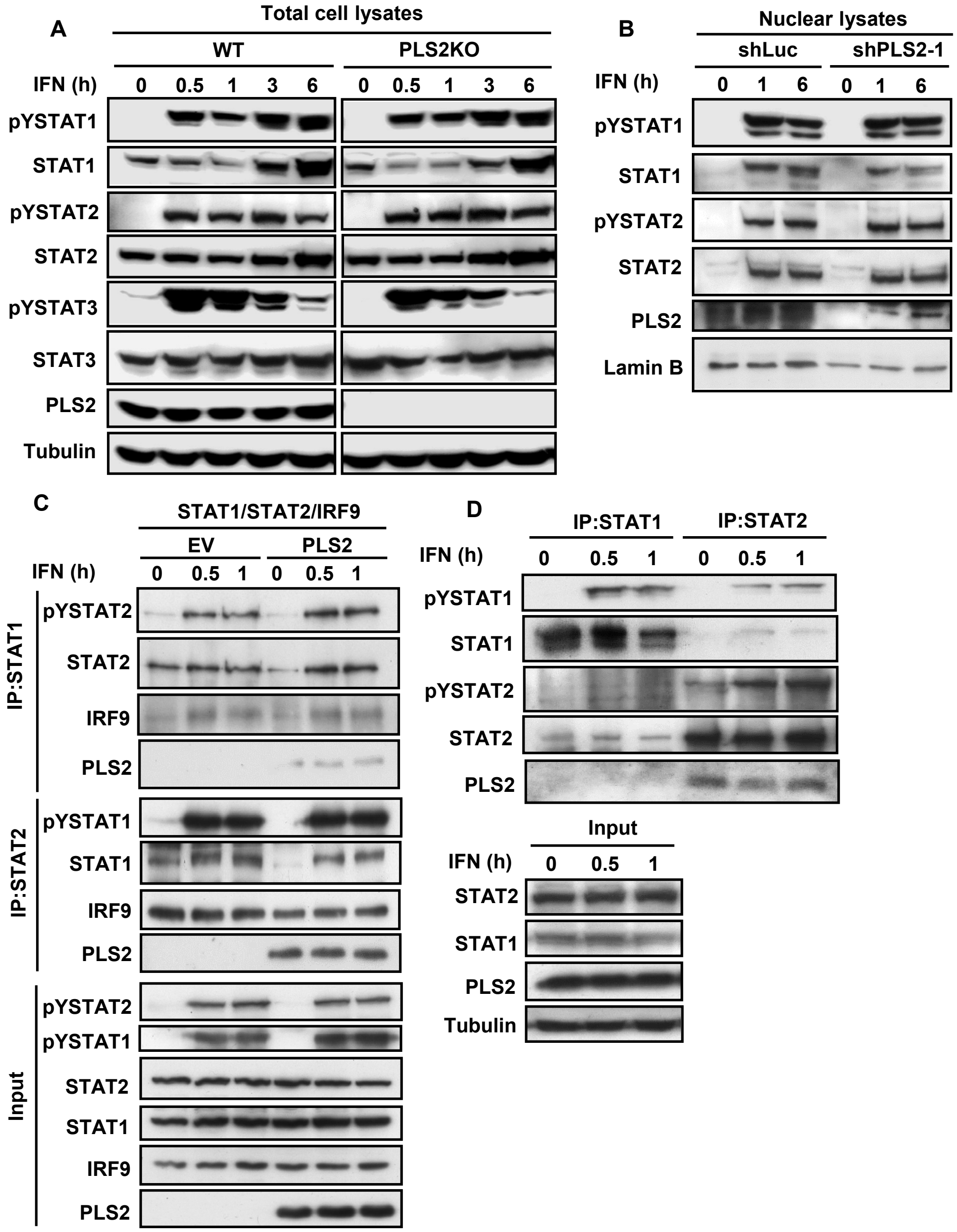

Figure S6. PLSCR2 does not affect the activation or nuclear translocation of STATs nor does it affect ISGF3 assembly. (A) WT or PLS2KO ML-1 cells were treated with mouse IFN-α4 for the indicated times. Total cell lysates were subjected to immunoblotting with the indicated antibodies. (B) WT MEFs expressing luciferase-(shLuc) or PLSCR2-specific (shPLS2-1) shRNA were treated with mouse IFN-α4 for the indicated times. Nuclear lysates were subjected to immunoblotting using the indicated antibodies. (C) HEK293T cells were co-transfected with HA-tagged STAT1, STAT2, and IRF9 with empty vector (EV) or myc-PLS2 and then treated with human IFN-α2, followed by co-IP with anti-STAT1 or anti-STAT2 antibody and immunoblotting with the indicated antibodies. Total lysates were also subjected to immunoblotting as the input control. (D) ML-1 cells were stimulated with mouse IFN-α4 for the indicated times, followed by co-IP as in (A) and immunoblotting with antibody against PLSCR2.

Figure S7

```
mPLSCR2    1 --------------------------------------------------      0

YFV NS5    1 KLSEFGKAKGSRAIWYMWLGARYLEFEALGFLNEDHWASRENSGGGVEGI     50

mPLSCR2    1 -----------MEAPRSGTYL---PAGYAPQYPPAAVQGPPEHTGRPTFQ     36
                        :.|...|.:.   .||:..:...|.:....|       .
YFV NS5   51 GLQYLGYVIRDLAALEGGGFYADDTAGWDTRITEADLDDEQE-------I     93

mPLSCR2   37 TNYQVP----------QSGYPGPQASYTVSTSGHEGY---AATRLPIQNN     73
             .||..|          :..|............|.:.|   .:.|....:.
YFV NS5   94 LNYMSPHHRKLALAVMEMTYKNKVVKVLRPAPGGKAYMDVISRRDQRGSG    143

mPLSCR2   74 QTIVLA-NTQWMPAPPPILNCPPGLEYLNQIDQLLIHQQVELLE--VLTG    120
             |.:..| ||        |.|....|..:.:.:.::.||.|:..:  |||.
YFV NS5  144 QVVTYALNT--------ITNLKVQLIRMAEAEMVIHHQHVQDCDDTVLTK    185

mPLSCR2  121 FETNNKFEIKNSLGQMVYVAVEDTDCCTRNCCEASRPFTLRI---LDHLG    167
             .|.   :..::...::..:||...||..       ||...|.   |.||.
YFV NS5  186 LEA---WLAEHGCDRLKRMAVSGDDCVV-------RPIDDRFGLALSHLN    225

mPLSCR2  168 QEVMTLERPLKCSSCCFPCCLQEIEIQAPPG------VPIGYVTQTWHPC    211
                 .:.:..|..|          |.|...|      ||  :.:..:|
YFV NS5  226 ----AMSKVRKDIS----------EWQPSKGWDDWENVP--FCSHHFH--    257

mPLSCR2  212 LPKLTLQNDKRENVLKVVGPCVACTCCSDIDFEIKSLDEVTRIGKIT--K    259
               :|.|::.:|     :|.||             :..||:...|:::  .
YFV NS5  258 --ELQLKDGRR-----IVVPC-------------RDQDELVGRGRVSPGN    287

mPLSCR2  260 QW----SGCVKEAFTDSDNFGIQFPLDLEVKMKAVTLGACFLIDYMFFEG    305
             .|    :.|:.:|:.:                         :...|:|..
YFV NS5  288 GWMIKETACLSKAYAN-------------------------MWSLMYFHK    312

mPLSCR2  306 CE-------------------------------    307
             .:
YFV NS5  313 RDMRLLSLAVSSAVPTSWVPQGRTTWSVHGKGE    345
```

Figure S7. PLSCR2 does not share sequence similarity with YFV NS5. Pairwise sequence alignment was performed using the EMBOSS Needle to do analysis for mouse PLSCR2 (Q9DCW2) and YFV NS5 (O55841) (https://www.ebi.ac.uk/Tools/psa/emboss_needle/). The overall identity is 13.6% (59/433 aa) and similarity is 24.2% (105/433 aa).
